# Synthesis of optically active folded cyclic dimers and trimers

**DOI:** 10.3762/bjoc.21.124

**Published:** 2025-08-11

**Authors:** Ena Kumamoto, Kana Ogawa, Kazunori Okamoto, Yasuhiro Morisaki

**Affiliations:** 1 Department of Applied Chemistry for Environment, School of Biological and Environmental Sciences, Kwansei Gakuin University, 1 Gakuen Uegahara, Sanda, Hyogo 669-1330, Japan

**Keywords:** circularly polarized luminescence, oligomer, [2.2]paracyclophane, planar chirality

## Abstract

Optically active higher-ordered structures, such as one-handed helical and propeller-shaped structures, can be constructed by folding the π-conjugated system using [2.2]paracyclophane as the chiral crossing unit, leading to circularly polarized luminescence (CPL) properties. Chiral cyclic dimers and trimers were synthesized using planar chiral [2.2]paracyclophane-containing enantiopure ribbon-shaped compounds as the chiral monomers. Unicursal π-conjugated systems were folded at the [2.2]paracyclophane units, and exhibited good photoluminescence quantum efficiencies and CPL anisotropy factors. Opposite chiroptical properties were observed between the dimer and trimer, despite the same absolute configuration of the planar chiral [2.2]paracyclophane units, which was reproduced by theoretical studies.

## Introduction

Cyclophane is a general term for cyclic compounds with at least one aromatic ring in the main chain backbone [1]. Cyclophane compounds have long been known; [2.2]paracyclophane was first reported as a cyclic dimer of paraxylylene by Brown et al. in 1949 [2]. In 1951, Cram et al. reported the practical synthesis of [2.2]paracyclophanes via Wurtz-type intramolecular cyclization [3]. [2.2]Paracyclophane has a molecular structure in which two benzene rings are stacked face-to-face with ethylene chains at the para positions. Various studies have been conducted on their reactivities and physical properties derived from their unique molecular structure with stacked π-electron clouds [1,4–6]. The distance between benzene rings in [2.2]paracyclophane is extremely short (2.8–3.1Å), and thus the rotational motion of benzene rings is completely suppressed; therefore, planar chirality without chiral centers [7] appears by introducing substituent(s) at appropriate position(s) on the benzene rings [8]. Enantiopure planar chiral [2.2]paracyclophanes have been used as chiral auxiliaries and chiral ligands for transition metals in the fields of organic and organometallic chemistry [9–20]. In 2012, enantiopure [2.2]paracyclophane was used as a chiral monomer to prepare optically active conjugated polymers and cyclic trimers [21], in which π-electron systems were stacked to form zigzag and triangular structures, respectively. The conjugated polymers and cyclic trimers exhibited circularly polarized luminescence (CPL) [22–25] with high photoluminescence (PL) quantum efficiency (Φ_PL_) and anisotropy. Additionally, the π-stacked structure of [2.2]paracyclophane can be applied at a crossing point. By folding the π-conjugated system using [2.2]paracyclophane as the chiral crossing unit, optically active higher-ordered structures, such as one-handed helical [26–27] and propeller-shaped structures [28–30], can be constructed, leading to the excellent CPL behaviors. Recently, planar chiral [2.2]paracyclophane-containing cyclic molecules have been received attention; for examples, the one-handed double helical compounds [31–34] and chiral nanohoops [35–36] emitting circularly polarized fluorescence have been reported. In this study, enantiopure ribbon-shaped compounds based on planar chiral tetrasubstituted [2.2]paracyclophane were used as chiral monomers, and optically active cyclic dimers and trimers, in which π-conjugated systems were folded in two and three places, respectively, were synthesized. Planar chiral [2.2]paracyclophane served as crosspoints to construct unicursal cyclic π-conjugated structures. The synthetic procedures and optically properties were investigated.

## Results and Discussion

**Scheme 1:**
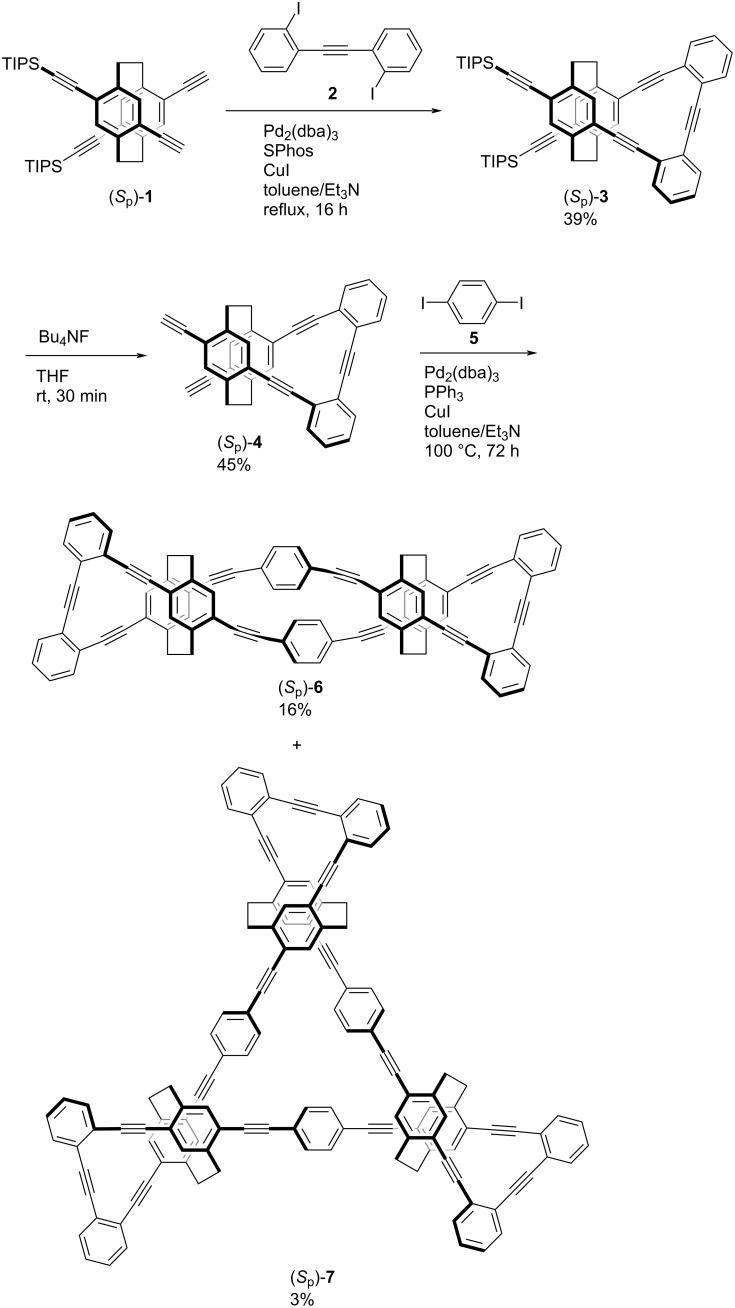
Synthesis of cyclic dimer (*S*_p_)-**6** and trimer (*S*_p_)-**7**.

Scheme 1 illustrates the synthetic routes to optically active cyclic dimer and trimer based on planar chiral tetrasubstituted [2.2]paracyclophane. Bis-(*para*)-pseudo-*ortho*-typed [15] [2.2]paracyclophane (*S*_p_)-**1** was prepared according to a literature’s procedure [31]. The Sonoghashira–Hagihara cross-coupling [37–38] of (*S*_p_)-**1** with diiodotolane **2** afforded the corresponding ribbon-shaped compound (*S*_p_)-**3** in 39% isolated yield. Triisopropylsilyl (TIPS) groups in (*S*_p_)-**3** were removed using Bu_4_NF to afford diyne (*S*_p_)-**4** as a monomer in 45% isolated yield. The reaction of (*S*_p_)-**4** with diiodobenzene **5** using a Pd_2_(dba)_3_/PPh_3_/CuI catalytic system in toluene and Et_3_N under diluted conditions (monomer concentration = approximately 1.3 × 10^−3^ M) was performed. The corresponding cyclic dimer (*S*_p_)-**6** and trimer (*S*_p_)-**7** were detected mainly by thin-layer chromatography (TLC) and separated roughly using simple SiO_2_ column chromatography. In addition, they were purified using a recyclable high-performance liquid chromatography (HPLC) to remove unidentified impurities to obtain (*S*_p_)-**6** and (*S*_p_)-**7** in 16% and 3% isolated yields, respectively.

**Figure 1:**
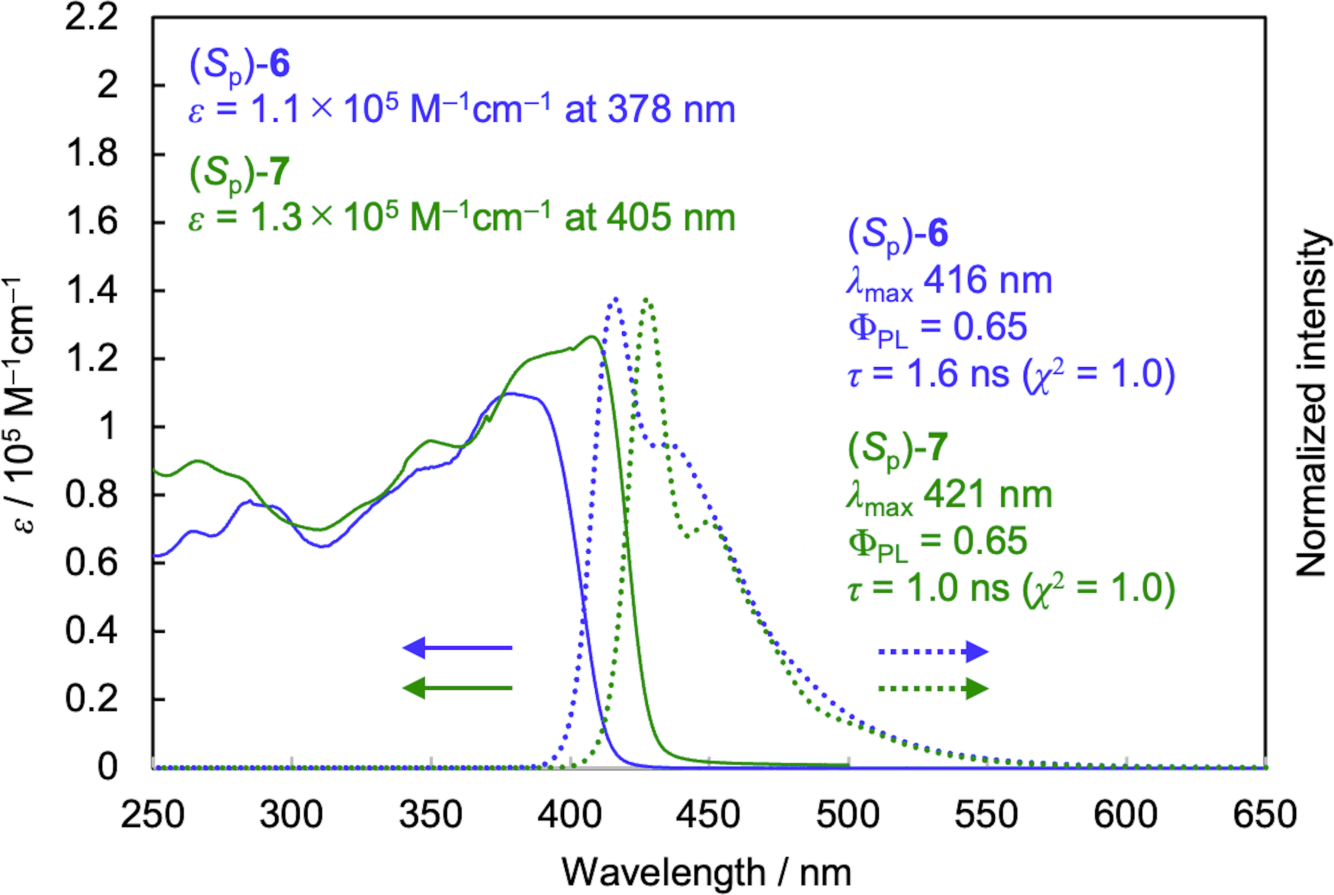
UV–vis and PL spectra of (*S*_p_)-**6** and (*S*_p_)-**7** in CHCl_3_ (1.0 × 10^−5^ M). Excitation wavelength 370 nm and 378 nm for (*S*_p_)-**6** and (*S*_p_)-**7**, respectively.

The ultraviolet–visible (UV–vis) absorption spectra and normalized photoluminescence (PL) spectra of (*S*_p_)-**6** and (*S*_p_)-**7** in diluted CHCl_3_ solutions (1.0 × 10^−5^ M) are depicted in Figure 1. The absorption bands are derived from the π–π* transitions of a phenylene–ethynylene conjugation system. The spectra of (*S*_p_)-**6** and (*S*_p_)-**7** are similar, and the absorption peak top of (*S*_p_)-**7** is red-shifted compared with that of (*S*_p_)-**6** because of the bent structure of the *p*-phenylene–ethynylene moieties in (*S*_p_)-**6** and extended π-conjugation of (*S*_p_)-**7**. Such a red-shift of a UV–vis absorption spectrum has been observed in previously reported [2.2]paracyclophane-based cyclic oligomers [39].

CHCl_3_ solutions (1.0 × 10^−5^ M) of (*S*_p_)-**6** and (*S*_p_)-**7** were photo-excited around their absorption peak maxima, and both oligomers emitted blue fluorescence as shown in Figure 1. Their PL spectra with vibrational structures were identical, and the PL quantum efficiencies (Φ_PL_) of both (*S*_p_)-**6** and (*S*_p_)-**7** were estimated to be 0.65. Their PL of the cast films fabricated from the toluene solutions were also measured, and weak and inefficient luminescence were observed because of the aggregation-caused PL quenching. The PL lifetimes of (*S*_p_)-**6** and (*S*_p_)-**7** in the CHCl_3_ solutions were measured at each PL peak maximum; and the PL decay curves (Figure S17, Supporting Information File 1) were fitted with the single exponential function. The lifetimes (*τ*) were estimated to be 1.6 ns and 1.0 ns, respectively.

**Figure 2:**
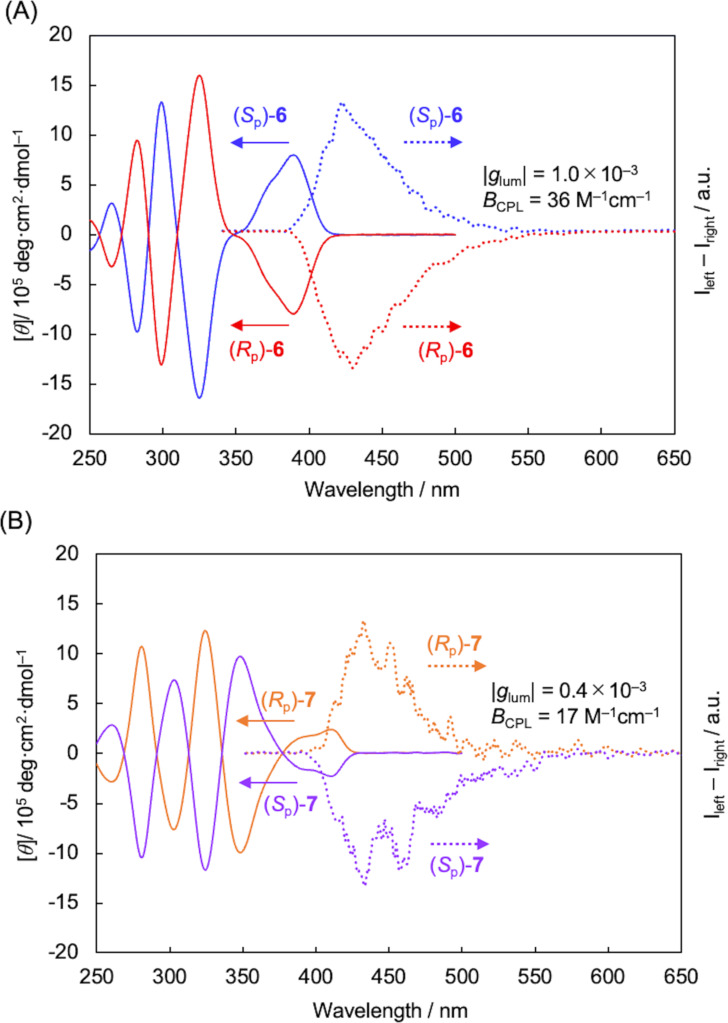
(A) UV, CD, PL, and CPL spectra of (*S*_p_)- and (*R*_p_)-**6** in CHCl_3_ (1.0 × 10^−5^ M). (B) UV, CD, PL, and CPL spectra of (*S*_p_)- and (*R*_p_)-**7** in CHCl_3_ (1.0 × 10^−5^ M). Excitation wavelength for CPL of both (*S*_p_)-**6** and (*S*_p_)-**7** = 300 nm.

Circular dichroism (CD) and CPL spectra of (*S*_p_)-**6** and (*S*_p_)-**7** were obtained in CHCl_3_ solutions (1.0 × 10^−5^ M), and the spectra are shown in Figure 2. As illustrated in Figure 2A, the mirror-image Cotton effect of (*S*_p_)- and (*R*_p_)-**6** was observed throughout the absorption band, and the absolute molar ellipticity |[θ]| reached on the order of 10^6^. Intense CPL signals were also obtained, and the |*g*_lum_| value [23–25] was estimated as 1.0 × 10^−3^. The *g*_lum_ plots of **6** and **7** are shown in Figure S18A, Supporting Information File 1. Figure 2B shows the CD and CPL spectra of (*S*_p_)- and (*R*_p_)-**7**. Mirror-image CD signals were observed, and the absolute molar ellipticity (|[θ]|) was smaller than that of **6**. The first Cotton effect of (*S*_p_)-**7** was negative, in contrast to that of (*S*_p_)-**6**. The sign of the first Cotton effect was consistent with that of CPL signal; the CPL sign of (*S*_p_)-**7** was also negative. Thus, the CD and CPL signs of (*S*_p_)-**6** and (*S*_p_)-**7** were opposite, despite the planar chiral [2.2]paracyclophane units having the same absolute configuration. The |*g*_lum_| value of **7** was 0.4 × 10^−3^ (Figure S18A, Supporting Information File 1), which was lower than that of **6**. In the case of the film states of **6** and **7**, low PL brightness and intermolecular random orientation of fluorophores resulted in noisy CPL signals.

**Figure 3:**
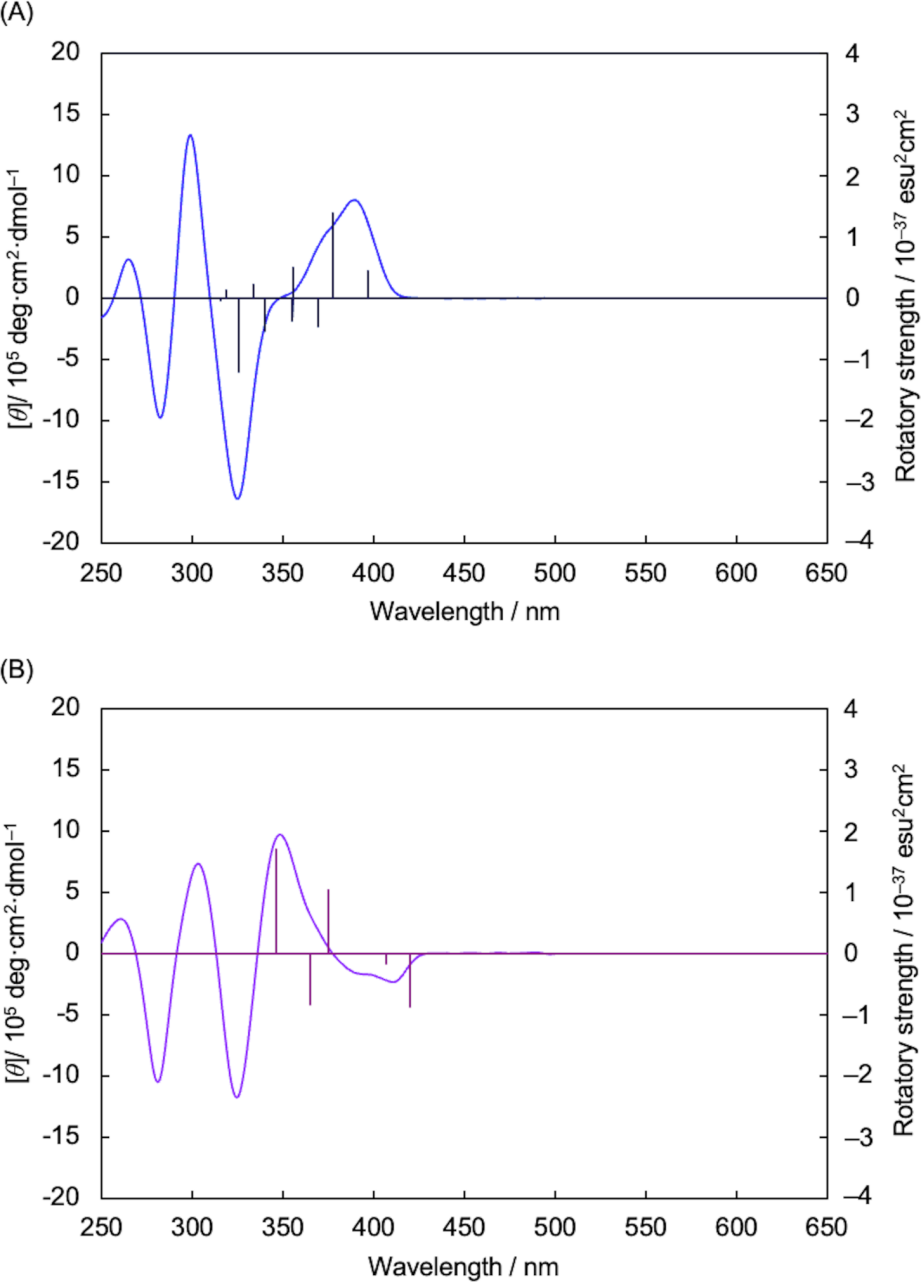
(A) CD spectrum of (*S*_p_)-**6** in CHCl_3_ (1.0 × 10^−5^ M), and the plot of the calculated rotatory strengths (TD-MN15/6-31G(d)//MN15/6-31G(d)). (B) CD spectrum of (*S*_p_)-**7** in CHCl_3_ (1.0 × 10^−5^ M), and the plot of the calculated rotatory strengths (TD-MN15/6-31G(d)//MN15/6-31G(d)).

The CD spectra were simulated by time-dependent density functional theory (TD-DFT) calculations (TD-MN15/6-31G(d)//MN15/6-31G(d). The calculated rotatory strengths of (*S*_p_)-**6** and (*S*_p_)-**7** were plotted with their observed CD spectra in Figure 3A and 3B, respectively, and the calculated CD spectra are shown in Supporting Information File 1, Figure S19A and S19B, respectively. Thus, the rotatory strengths corresponded well the observed CD spectra (Figure 3A and 3B). The signs of the simulated CD spectrum of (*S*_p_)-**6** were positive and negative from the long wavelength to the short wavelength (Figure S19A, Supporting Information File 1), which reproduced the experimental CD spectrum of (*S*_p_)-**6**. In addition, the signs of the experimental CD spectrum of (*S*_p_)-**7**, negative and positive from the long wavelength to the short wavelength, were reproduced using the simulated CD spectrum (Figure S19B, Supporting Information File 1).

**Figure 4:**
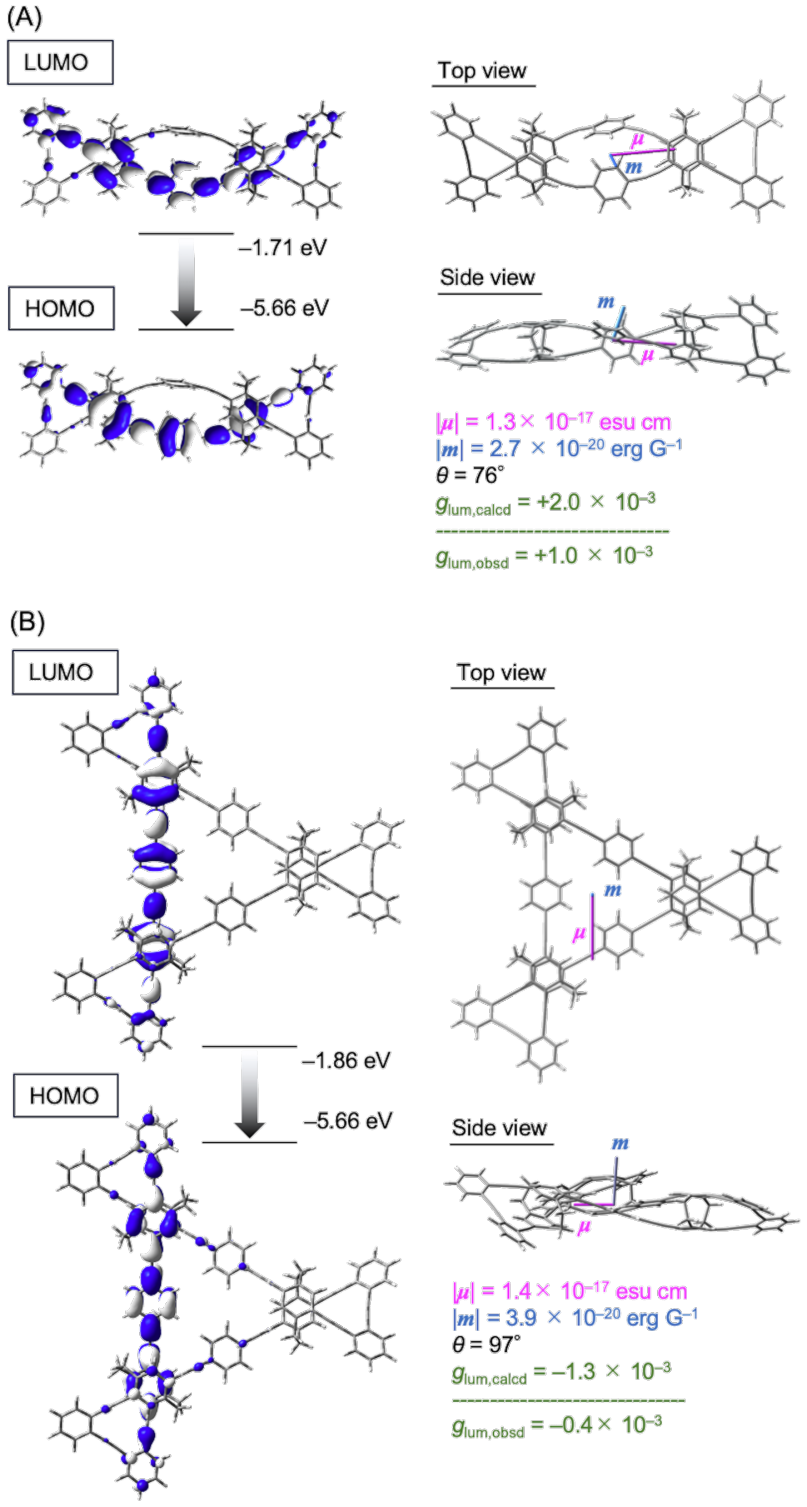
Molecular orbitals and simulated CPL profiles in the S_1_ states of (A) (*S*_p_)-**6** and (B) (*S*_p_)-**7** (TD-MN15/6-31G(d)).

Molecular orbitals of (*S*_p_)-**6** and (*S*_p_)-**7** in their ground and excited states were calculated using DFT and TD-DFT calculations (Figures S20–S23, Supporting Information File 1). In both molecules, the orbitals were localized to part of the π-conjugation systems rather than the whole system in the ground and excited states due to the twisted structures by the π-stacked [2.2]paracyclophane moieties. The CPL behaviors of (*S*_p_)-**6** and (*S*_p_)-**7** were investigated by TD-DFT calculations; namely, the electric transition dipole moment (μ), magnetic transition dipole moment (*m*), and the angle (θ) between μ and m in the S_1_ states were simulated. The *g*_lum_ value was theoretically calculated by the following equation: *g*_lum_ = 4|μ||*m*|cosθ/(|μ|^2^ + |*m*|^2^) ≈ 4|*m*|cosθ/|μ|. The molecular orbitals of (*S*_p_)-**6** and (*S*_p_)-**7** in the S_1_ states involved in CPL are illustrated in Supporting Information File 1, Figure 4A and 4B. The molecular orbitals of (*S*_p_)-**6** were localized in one curved π-electron system, and the μ extended along the long axis of the molecule. The value of the angle θ of (*S*_p_)-**6** was calculated to be 76°, which supported the positive CPL sign. The molecular orbitals of (*S*_p_)-**7** in the S_1_ states are shown in Figure 4B. The orbitals are linearly localized in the portion of the extended π-electron system rather than the entire molecule. Therefore, the elongation of the μ could not be suppressed, and thus a lower |*g*_lum_| value was obtained. The angle θ of (*S*_p_)-**7** was 97°; it is consistent with the negative CPL sign. As described above, the experimental CD and CPL signs of (*S*_p_)-**6** and (*S*_p_)-**7** were reproduced by the TD-DFT calculations. Molecular orbitals of (*S*_p_)-**6** involved in the CPL are obviously curved and twisted, resulting in the opposite chiroptical signs. Twisted chirality is known to result in CPL of π-conjugated molecules; for example, twisted anthracene is a good CPL emitter [40–47]; the more the anthracene is twisted, the better the CPL properties [46]. As for the π-stacked cyclic oligomers, the trimers and tetramers exhibited good CPL properties, whereas the cyclic dimer consisting of curved π-electron systems was not a good CPL emitter [39]. Thus, one of our next targets is to clarify experimentally and theoretically the CPL behavior of linear π-electron systems such as *p*-phenylene-ethynylene, when they are curved and twisted.

## Conclusion

In summary, two types of optically active oligomers using planar chiral tetrasubstituted [2.2]paracyclophane as chiral crossing units were prepared, in which π-extended conjugated systems were folded in either two or three places. The oligomers exhibited good optical properties such as PL and CPL properties with good Φ_PL_ values (>0.6) and |*g*_lum_| values of the ordered of 10^–4^–10^–3^. The chiroptical properties of dimers and trimers showed opposite Cotton effects and CPL signals, despite the same absolute configuration of the planar chiral [2.2]paracyclophane crossing points. The π-conjugation system of the dimer that exhibited CPL was highly curved and twisted, which caused the different chiroptical properties.

## Experimental

### General

^1^H and ^13^C NMR spectra were recorded on a JEOL JNM ECZ-500R instrument at 500 and 125 MHz, respectively. Samples were analyzed in CDCl_3_, and the chemical shift values were expressed relative to Me_4_Si as an internal standard. Analytical thin-layer chromatography (TLC) was performed with silica gel 60 Merck F_254_ plates. Column chromatography was performed with silica gel 60N (spherical neutral). Recyclable preparative high-performance liquid chromatography (HPLC) was carried out on a Japan Analytical Industry Model LC918R (JAIGEL 1H and 2H gel-permeation columns) using CH_2_Cl_2_ as an eluent. Recyclable chiral chromatography (HPLC) was carried out on a YMC LC Forte/R (Chiralpak^®^ IA column). High-resolution mass spectra (HRMS) was obtained on a Bruker Daltonics microTOF II spectrometer (APCI) by using sodium formate and tuning mix as internal standard or on a JEOL JMS-S3000 spectrometer for matrix-assisted desorption/ionization (MALDI) with *trans*-2-[3-(4-*tert*-butylphenyl)-2-methyl-2-propenylidene]malononitrile (DCTB) as a matrix. UV–vis absorption spectra were recorded on a JASCO V-730 spectrophotometer, and samples were analyzed in CHCl_3_ at room temperature. Photoluminescence (PL) spectra were recorded on a JASCO FP-8500 spectrofluorometer, and samples were analyzed in CHCl_3_ at room temperature. Absolute PL quantum efficiency was calculated on a JASCO FP-8500 with an ILF-835 integrating sphere. The PL lifetime measurement was performed on a Hamamatsu Photonics Quantaurus-Tau fluorescence lifetime spectrometer system. Specific rotations ([*α*]_D_*^t^*) were measured with a HORIBA SEPA-500 polarimeter: concentration “*c*” is g/dL. Circular dichroism (CD) spectra were recorded on a JASCO J-1500 spectropolarimeter with CHCl_3_ as a solvent at room temperature. Circularly polarized luminescence (CPL) spectra were recorded on a JASCO CPL-300 with CHCl_3_ as a solvent at room temperature.

### Materials

Commercially available compounds used without purification are as follows: Pd_2_(dba)_3_, SPhos (2-dicyclohexylphosphino-2',6'-dimethoxybiphenyl), PPh_3_, CuI, Bu_4_NF, MeOH, dehydrated THF, dehydrated toluene, and 1,4-diiodobenzene (**5**). Et_3_N was purchased and distilled over KOH. (*S*_p_)-**1** [31], (*R*_p_)-**1** [31], and **2** [48] were prepared as described in the literature.

### Synthesis of (*S*_p_)-**3**

A mixture of (*S*_p_)-**1** (49.6 mg, 0.081 mmol), **2** (25.3 mg, 0.059 mmol), Pd_2_(dba)_3_ (6.1 mg, 0.0067 mmol), SPhos (5.6 mg, 0.014 mmol), CuI (1.8 mg, 0.0095 mmol), toluene (40 mL) and Et_3_N (40 mL) was placed in a round-bottom flask equipped with a magnetic stirring bar. After degassing the reaction mixture several times, the mixture was heated at reflux temperature for 16 h. After the reaction mixture was cooled to room temperature, the solvent was removed with a rotary evaporator. The residue was purified by column chromatography on SiO_2_ (CHCl_3_/hexane = 1/4 v/v as an eluent) and by recyclable HPLC (CH_2_Cl_2_ as an eluent) to afford (*S*_p_)-**3** (24.6 mg, 0.031 mmol, 39%) as a light yellow solid. *R*_f_ = 0.73 (CHCl_3_/hexane = 1:2 v/v); ^1^H NMR (CDCl_3_, 500 MHz) δ 1.20 (s, 42H), 2.97–3.08 (m, 4H), 3.49–3.53 (m, 4H), 7.06 (s, 2H), 7.21 (s, 2H), 7.37–7.40 (m, 4H), 7.60–7.62 (m, 2H), 7.70–7.73 (m, 2H) ppm; ^13^C NMR (CDCl_3_, 125 MHz) δ 11.48, 18.84, 32.32, 32.57, 92.38, 93.65, 93.81, 95.72, 105.99, 125.16, 125.33, 125.42, 125.91, 128.07, 128.27, 131.99, 133.24, 134.65, 135.30, 141.30, 142.58 ppm: HRMS (APCI+) (*m*/*z*): [M + H]^+^ calcd. for C_56_H_62_Si_2_, 791.4463; found, 791.4472; [α]_D_^25^ –203.25 (*c* 0.04, CHCH_3_).

(*R*_p_)-**3** was obtained by the same procedure of (*S*_p_)-**3**. HRMS (APCI+) (*m*/*z*): [M + H]^+^ calcd. for C_56_H_62_Si_2_, 791.4463; found, 791.4442; [α]_D_^25^ = +199.73 (*c* 0.04, CHCH_3_).

### Synthesis of (*S*_p_)-**4**

(*S*_p_)-**3** (43.0 mg, 0.054 mmol) was dissolved in THF (2 mL), followed by the addition of Bu_4_NF (1.0 M in THF solution, 0.11 mL). The reaction was carried out at room temperature for 30 min, and then H_2_O was added to the reaction mixture. The organic layer was extracted three times with CH_2_Cl_2_, and the combined organic layers were washed with saturated aqueous NaHCO_3_ and brine. After drying over MgSO_4_ and filtration, the solvent was removed under reduced pressure. The residue was purified by recyclable HPLC (CH_2_Cl_2_ as an eluent) to afford (*S*_p_)-**4** (11.8 mg, 0.025 mmol, 45%) as a light yellow solid. *R*_f_ = 0.43 (CHCl_3_/hexane = 1:2 v/v). ^1^H NMR (CDCl_3_, 500 MHz) δ 2.98–3.09 (m, 4H), 3.39 (s, 2H), 3.43–3.55 (m, 4H), 7.09 (s, 2H), 7.21 (s, 2H), 7.37–7.41 (m, 4H), 7.62–7.65 (m, 2H), 7.70–7.73 (m, 2H) ppm; ^13^C NMR (CDCl_3_, 125 MHz) δ 32.31, 32.43, 81.96, 83.04, 92.45, 93.34, 93.94, 124.30, 125.18, 125.98, 126.02, 128.25, 128.37, 132.22, 133.29, 135.15, 135.42, 141.47, 142.64 ppm; HRMS (APCI+) (*m*/*z*): [M + H]^+^ calcd. for C_38_H_22_, 479.1794; found, 479.1774. [α]_D_^25^ −278.90 (*c* 0.04, CHCH_3_).

(*R*_p_)-**4** was obtained by the same procedure of (*S*_p_)-**4**. HRMS (APCI+) (*m*/*z*): [M + H]^+^ calcd. for C_38_H_22_, 479.1794; found, 479.1816. [α]_D_^25^ = +278.68 (*c* 0.04, CHCH_3_).

### Synthesis and isolation of cyclic dimer (*S*_p_)-**6** and trimer (*S*_p_)-**7**

A mixture of (*S*_p_)-**4** (18.2 mg, 0.038 mmol), 1,4-diiodobenzene (**5**, 13.2 mg, 0.040 mmol), Pd_2_(dba)_3_ (9.0 mg, 0.0098 mmol), PPh_3_ (12.2 mg, 0.047 mmol), CuI (2.3 mg, 0.012 mmol), toluene (15 mL) and Et_3_N (15 mL) was placed in a round-bottom flask equipped with a magnetic stirring bar. After degassing the reaction mixture several times, the mixture was heated at reflux temperature for 72 h. After the reaction mixture was cooled to room temperature, the solvent was removed with a rotary evaporator. The residue was purified by column chromatography on SiO_2_ (hexane/ethyl acetate = 4:1 v/v as an eluent). The first and second fractions included mainly dimer (*S*_p_)-**6** and trimer (*S*_p_)-**7**, respectively. Dimer (*S*_p_)-**6** (6.8 mg, 0.0062 mmol, 16%) was isolated from the first fraction by recyclable HPLC (CH_2_Cl_2_ as an eluent) as a light yellow solid. Trimer (*S*_p_)-**7** was isolated from the second fraction by recyclable HPLC (CH_2_Cl_2_ as an eluent). Further purification of (*S*_p_)-**7** was carried out using chiral HPLC (CH_2_Cl_2_/hexane = 5:5 v/v as an eluent) to obtain (*S*_p_)-**7** (2.0 mg, 0.0012 mmol, 3%) as a yellow solid. Enantiomers were obtained by the same procedure from (*R*_p_)-**4**.

(*S*_p_)-**6**. *R*_f_ = 0.40 (hexane/ethyl acetate = 4:1 v/v). ^1^H NMR (CDCl_3_, 500 MHz) δ 3.12 (m, 8H), 3.46 (m, 4H), 3.60 (m, 4H), 7.23 (s, 8H), 7.31 (s, 8H), 7.38 (m, 8H), 7.64 (m, *J =* 8.59 Hz, 4H), 7.70 (m, *J =* 8.59 Hz, 4H) ppm; ^13^C{^1^H} NMR (CDCl_3_, 125 MHz) δ 29.8, 32.5, 32.8, 92.4, 92.7, 93.3, 93.7, 95.0, 123.5, 125.4, 125.8, 126.0, 128.2, 128.4, 131.5, 132.5, 133.1, 135.3, 136.7, 140.7, 141.7 ppm. HRMS (MALDI) (*m*/*z*): [M + Ag]^+^: calcd. for C_88_H_48_Ag, 1211.2802; found, 1211.2823. [α]_D_^25^ +386.63 (*c* 0.02, CHCl_3_).

(*R*_p_)-**6**. HRMS (MALDI) (*m*/*z*): [M + Ag]^+^calcd. for C_88_H_48_Ag 1211.2802; found, 1211.2848. [α]_D_^25^ –386.63 (*c* 0.02, CHCl_3_).

(*S*_p_)-**7**. *R*_f_ = 0.29 (hexane/ethyl acetate = 4/1 v/v). ^1^H NMR (CDCl_3_, 500 MHz) δ 3.10–3.18 (m, 12H), 3.60–3.66 (m, 12H), 7.23 (s, 6H), 7.28 (s, 6H), 7.45–7.47 (m, 12H), 7.70–7.71 (m, 6H), 7.76–7.79 (m, 18H) ppm; ^13^C{^1^H} NMR (CDCl_3_, 125 MHz) δ 32.60, 32.75, 91.31, 92.41, 93.47, 93.99, 94.29, 123.59, 125.14, 125.75, 125.91, 128.17, 128.27, 128.32, 131.70, 132.16, 133.24, 134.60, 135.59, 141.52, 142.25 ppm; HRMS (MALDI) (*m*/*z*): [M + Ag]^+^ calcd. for C_132_H_72_Ag 1763.4680; found, 1763.4729; [α]_D_^25^ = −739.17 (*c* 0.01, CHCl_3_).

(*R*_p_)-**7**. HRMS (MALDI) (*m*/*z*): [M + Ag]^+^ calcd. for C_132_H_72_Ag, 1763.4680; found, 1763.4647; [α]_D_^25^ +739.75 (*c* 0.01, CHCl_3_).

## Supporting Information

File 1 Statement of computational methods, NMR and HRMS spectra, PL decay curves, *g*_lum_ charts, calculated ECD spectra, and cartesian coordinates.

## Data Availability

All data that supports the findings of this study is available in the published article and/or the supporting information of this article.
