# Supplementary material for: Synthesis of optically active folded cyclic dimers and trimers
Source: Beilstein J Org Chem. 2025 Aug 11;21:1603–12. doi: 10.3762/bjoc.21.124 (PMC12362300; doi:10.3762/bjoc.21.124)
Supplement: File 1 — Statement of computational methods, NMR and HRMS spectra, PL decay curves, glum charts, calculated ECD spectra, and cartesian coordinates. [file Beilstein_J_Org_Chem-21-1603-s001.pdf]

BEILSTEIN JOURNAL OF ORGANIC CHEMISTRY

Supporting Information

for

Synthesis of optically active folded cyclic dimers and trimers

Ena Kumamoto, Kana Ogawa, Kazunori Okamoto and Yasuhiro Morisaki

Beilstein J. Org. Chem. **2025,** *21,* 1603–1612. doi:10.3762/bjoc.21.124

Statement of computational methods, NMR and HRMS spectra, PL decay curves, g_{lum} charts, calculated ECD spectra, and cartesian coordinates

License and Terms: This is a supporting information file under the terms of the Creative Commons Attribution License (https://creativecommons.org/licenses/by/4.0). Please note that the reuse, redistribution and reproduction in particular requires that the author(s) and source are credited and that individual graphics may be subject to special legal provisions.
The license is subject to the *Beilstein Journal of Organic Chemistry* terms and conditions: (https://www.beilstein-journals.org/bjoc/terms)

Computational methods

Density-functional theory (DFT) and time-dependent-DFT (TD-DFT) calculations [1-5] were carried out by the Gaussian 16 program package [6], with the 6-31G(d) [7-9] basis set for C and H atoms. Optimized geometries and their molecular orbitals in the ground and S_1 states were determined by DFT and TD-DFT calculation with the MN15 functional [10]. Cartesian coordinates of optimized structures are given in Tables S1–S4.

[1] M. E. Casida, C. Jamorski, K. C. Casida, D. R. Salahub, *J. Chem. Phys.* **1998**, *108*, 4439-4449.

[2] R. E. Stratmann, G. E. Scuseria, M. J. Frisch, *J. Chem. Phys.* **1998**, *109*, 8218-8224.

[3] R. Bauernschmitt, R. Ahlrichs, *Chem. Phys. Lett.* **1996**, 256, 454-464.

[4] C. Adamo, D. Jacquemin, *Chem. Soc. Rev.* **2013**, *42*, 845-856.

[5] C. Adamo, T. Le Bahers, M. Savarese, L. Wilbraham, G. García, R. Fukuda, M. Ehara, N. Rega, I. Ciofini, *Coord. Chem. Rev.* **2015**, *304–305*, 166-178.

[6] Gaussian 16, Revision B.01, M. J. Frisch, G. W. Trucks, H. B. Schlegel, G. E. Scuseria, M. A. Robb, J. R. Cheeseman, G. Scalmani, V. Barone, G. A. Petersson, H. Nakatsuji, X. Li, M. Caricato, A. V. Marenich, J. Bloino, B. G. Janesko, R. Gomperts, B. Mennucci, H. P. Hratchian, J. V. Ortiz, A. F. Izmaylov, J. L. Sonnenberg, D. Williams-Young, F. Ding, F. Lipparini, F. Egidi, J. Goings, B. Peng, A. Petrone, T. Henderson, D. Ranasinghe, V. G. Zakrzewski, J. Gao, N. Rega, G. Zheng, W. Liang, M. Hada, M. Ehara, K. Toyota, R. Fukuda, J. Hasegawa, M. Ishida, T. Nakajima, Y. Honda, O. Kitao, H. Nakai, T. Vreven, K. Throssell, J. A. Montgomery, Jr., J. E. Peralta, F. Ogliaro, M. J. Bearpark, J. J. Heyd, E. N. Brothers, K. N. Kudin, V. N. Staroverov, T. A. Keith, R. Kobayashi, J. Normand, K. Raghavachari, A. P. Rendell, J. C. Burant, S. S. Iyengar, J. Tomasi, M. Cossi, J. M. Millam, M. Klene, C. Adamo, R. Cammi, J. W. Ochterski, R. L. Martin, K. Morokuma, O. Farkas, J. B. Foresman, and D. J. Fox, Gaussian, Inc., Wallingford CT, 2016.

[7] M. M. Francl, W. J. Pietro, W. J. Hehre, J. S. Binkley, M. S. Gordon, D. J. DeFrees, J. A. Pople, *J. Chem. Phys.* **1982**, *77*, 3654-3665.

[8] P. C. Hariharan, J. A. Pople, *Theor. Chim. Acta* **1973**, *28*, 213-222.

[9] T. Clark, J. Chandrasekhar, G. W. Spitznagel, P. V. R. Schleyer, *J. Comput. Chem.* **1983**, *4*, 294-301.

[10] H. S. Yu, X. He, S. L. Li, D. G. Truhlar, *Chem. Sci.* **2016**, *7*, 5032-5051.

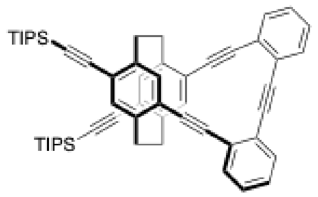

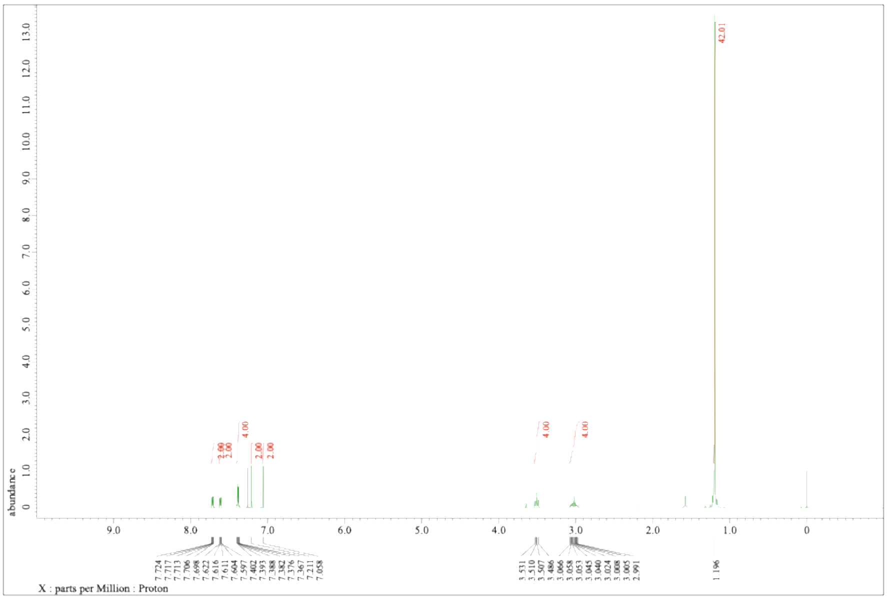

Figure S1: ^{1}H NMR ($CDCl_3$, 500 MHz) spectrum of (S_p)-**3**.

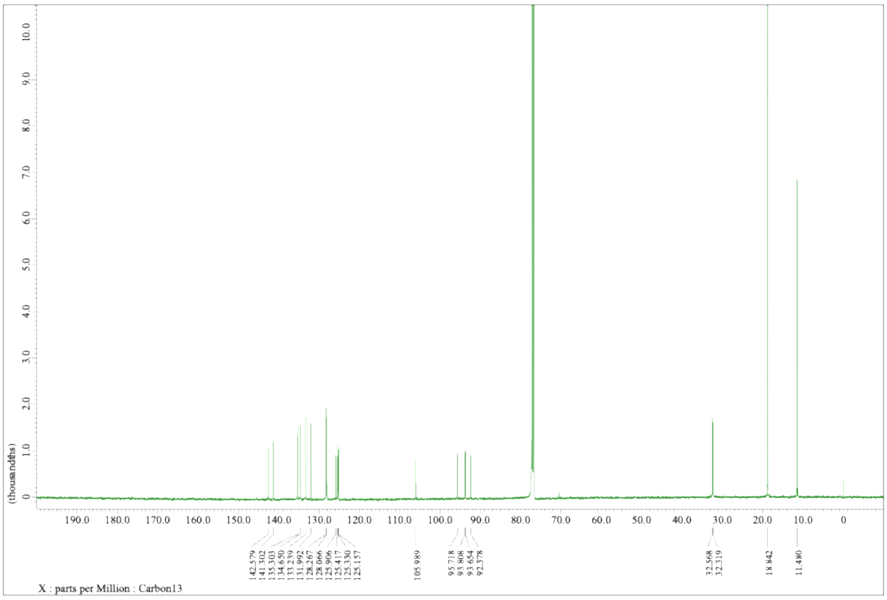

Figure S2: $^{13}C\{^{1}H\}$ NMR ($CDCl_3$, 125 MHz) spectrum of (S_p)-**3**.

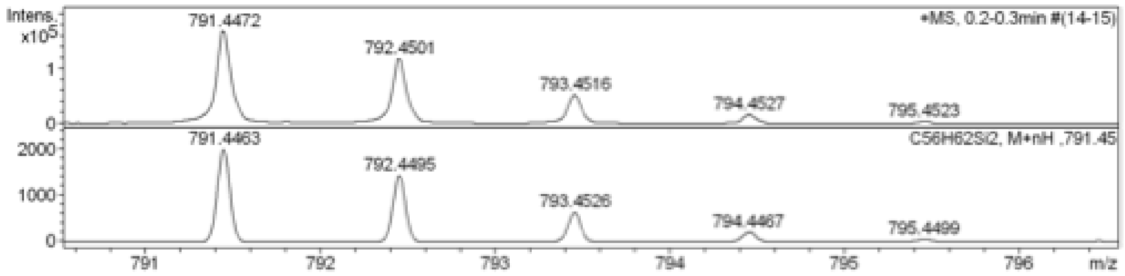

Figure S3: HRMS (APCI) spectra of (S_p)-**3**; upper and lower indicate experimental and theoretical mass spectra, respectively.

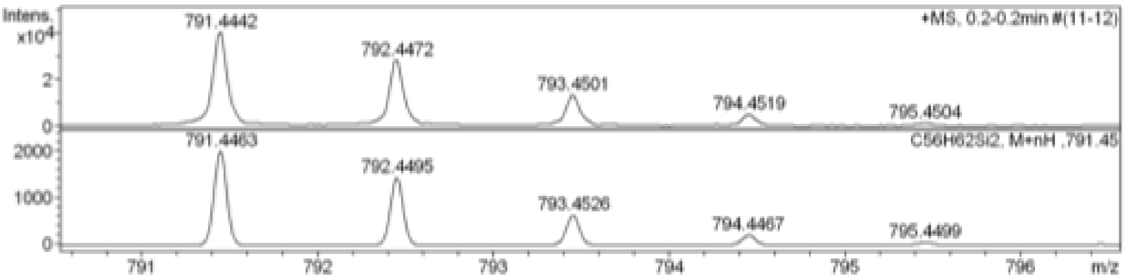

Figure S4: HRMS (APCI) spectra of (R_p)-**3**; upper and lower indicate experimental and theoretical mass spectra, respectively.

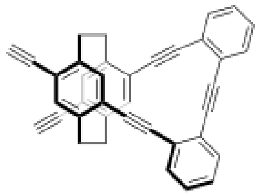

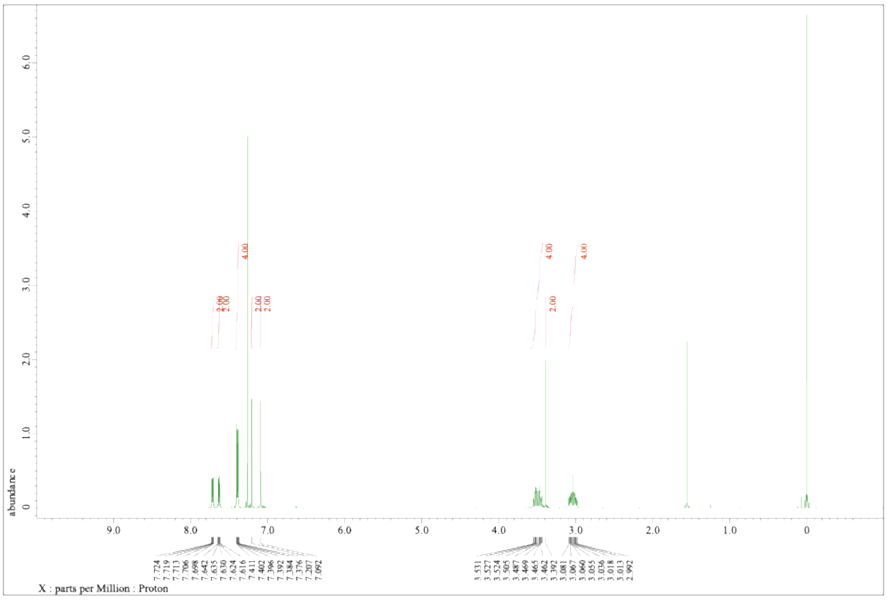

Figure S5: ^{1}H NMR ($CDCl_3$, 500 MHz) spectrum of (S_p)-**4**.

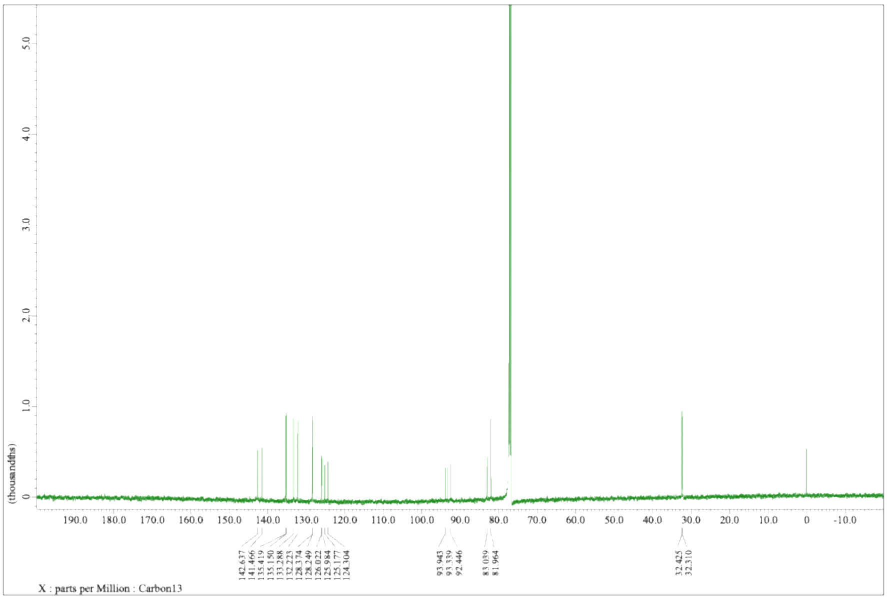

Figure S6: $^{13}C\{^{1}H\}$ NMR ($CDCl_3$, 125 MHz) spectrum of (S_p)-**4**.

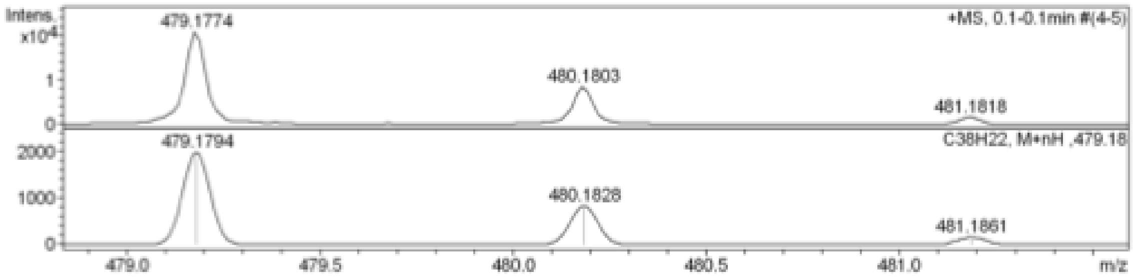

Figure S7: HRMS (APCI) spectra of (S_p)-**4**; upper and lower indicate experimental and theoretical mass spectra, respectively.

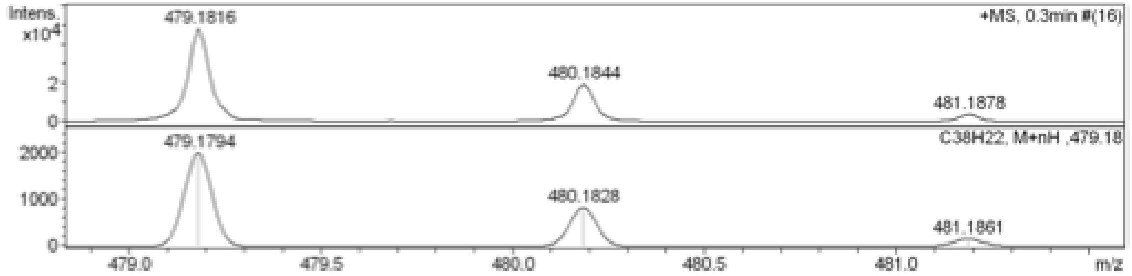

Figure S8: HRMS (APCI) spectra of (R_p)-**4**; upper and lower indicate experimental and theoretical mass spectra, respectively.

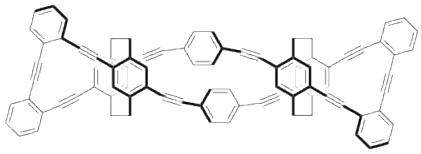

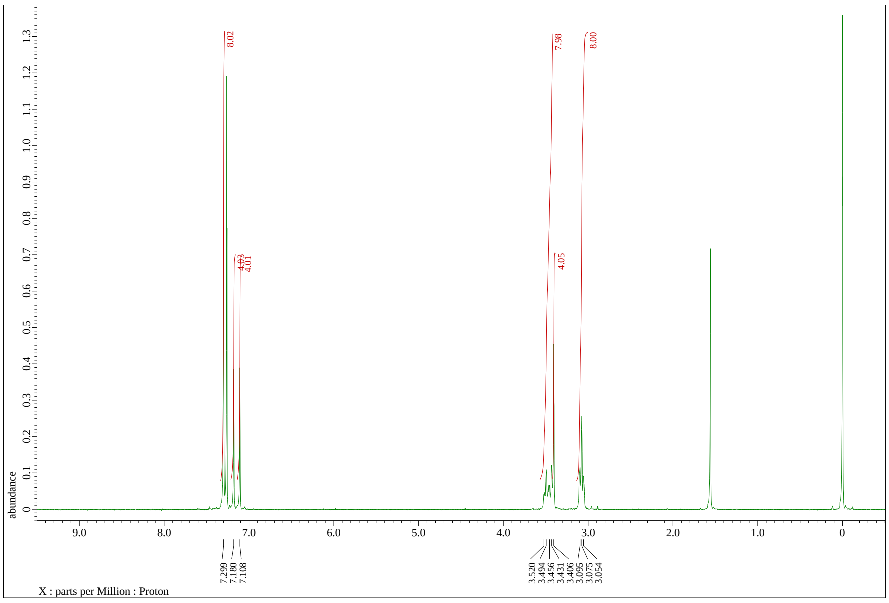

Figure S9: ^{1}H NMR ($CDCl_3$, 500 MHz) spectrum of (S_p)-**6**.

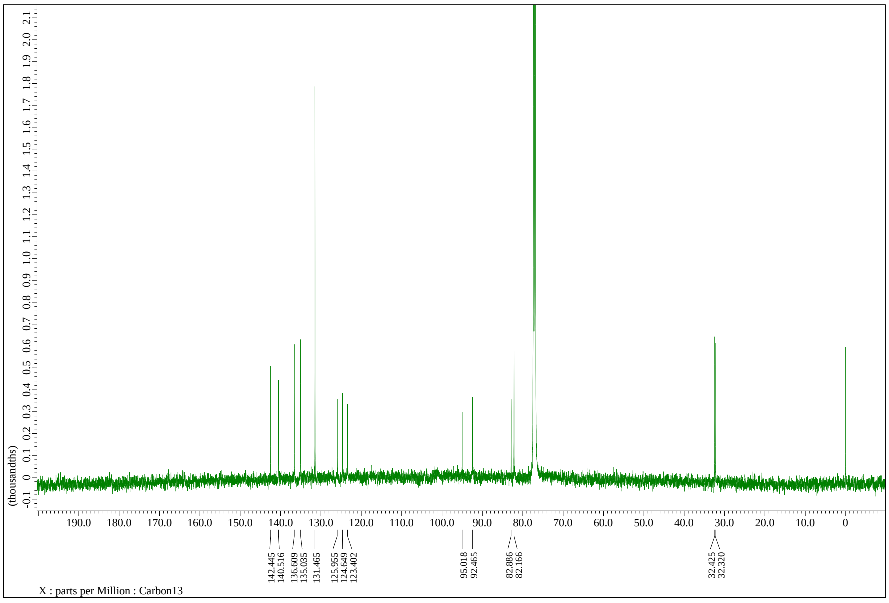

Figure S10: $^{13}C\{^{1}H\}$ NMR ($CDCl_3$, 125 MHz) spectrum of (S_p)-**6**.

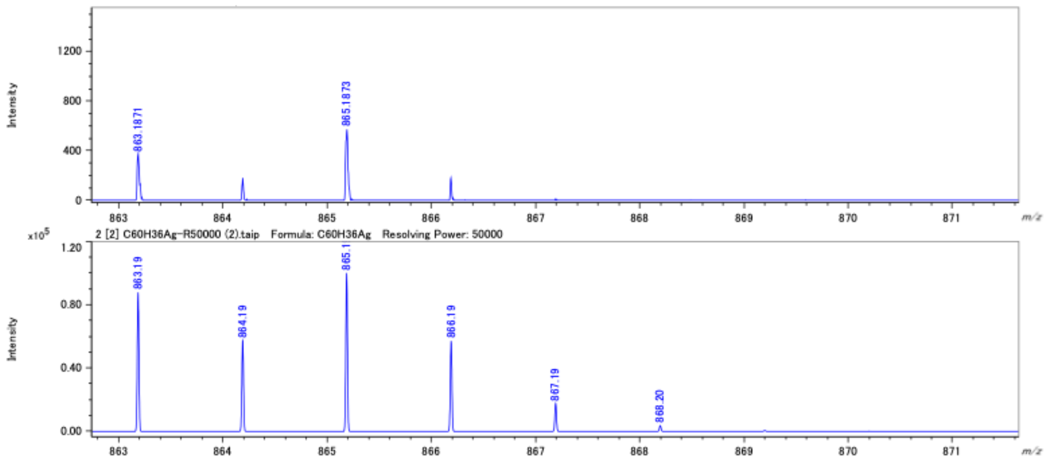

Figure S11: HRMS (MALDI) spectra of (S_p)-**6**; upper and lower indicate experimental and theoretical mass spectra, respectively.

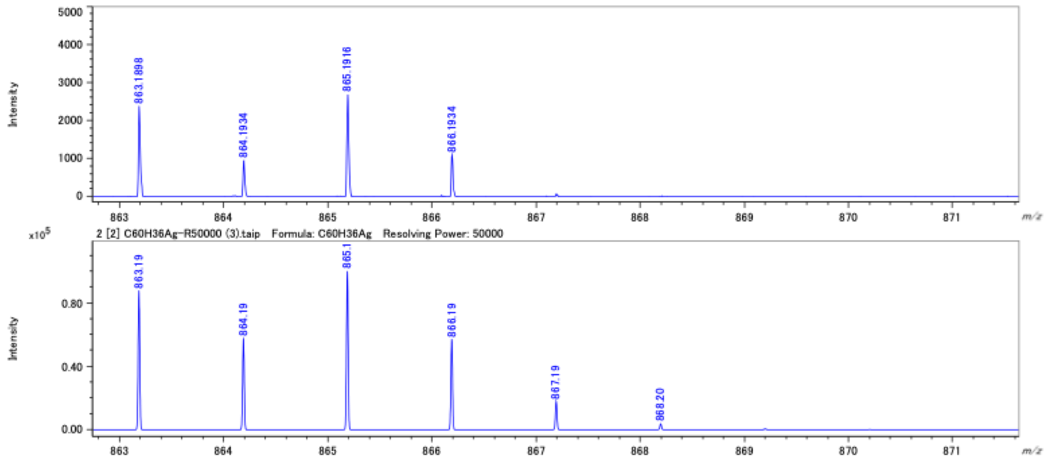

Figure S12: HRMS (MALDI) spectra of (R_p)-**6**; upper and lower indicate experimental and theoretical mass spectra, respectively.

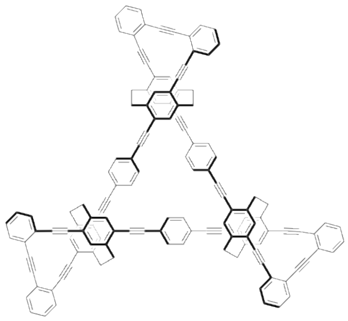

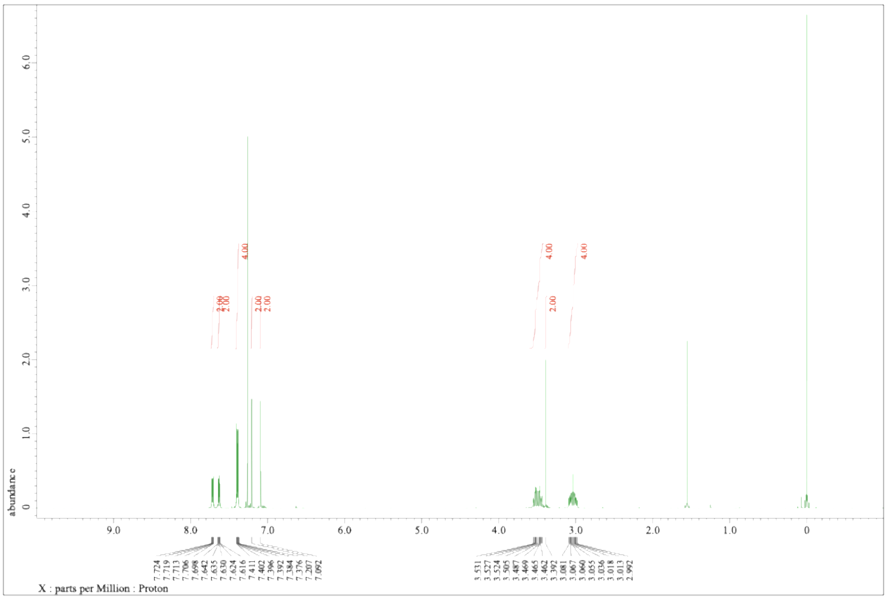

Figure S13: ^{1}H NMR (CDCl$_3$, 500 MHz) spectrum of (S_p)-**7**.

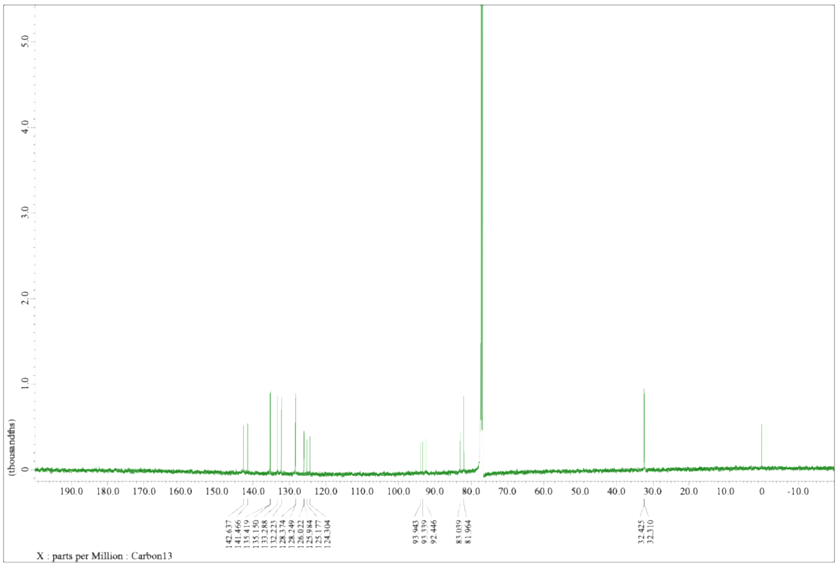

Figure S14: ^{13}C{^{1}H} NMR (CDCl$_3$, 125 MHz) spectrum of (S_p)-**7**.

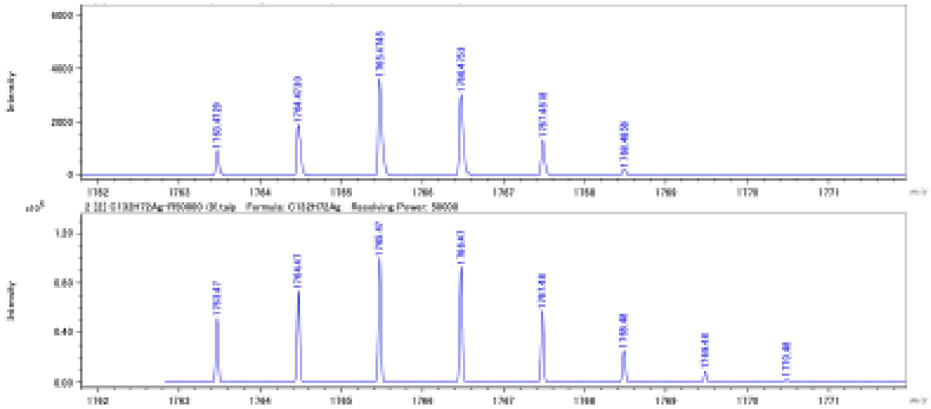

Figure S15: HRMS (MALDI) spectra of (S_p)-**7**; upper and lower indicate experimental and theoretical mass spectra, respectively.

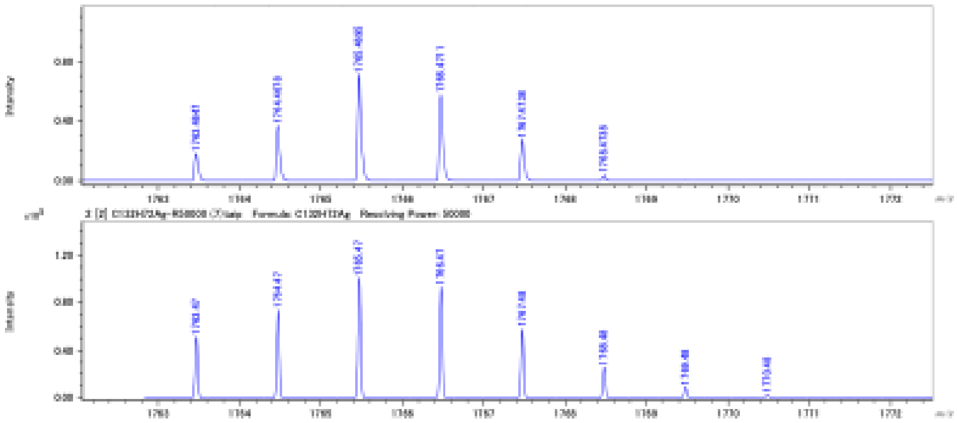

Figure S16: HRMS (MALDI) spectra of (R_p)-**7**; upper and lower indicate experimental and theoretical mass spectra, respectively.

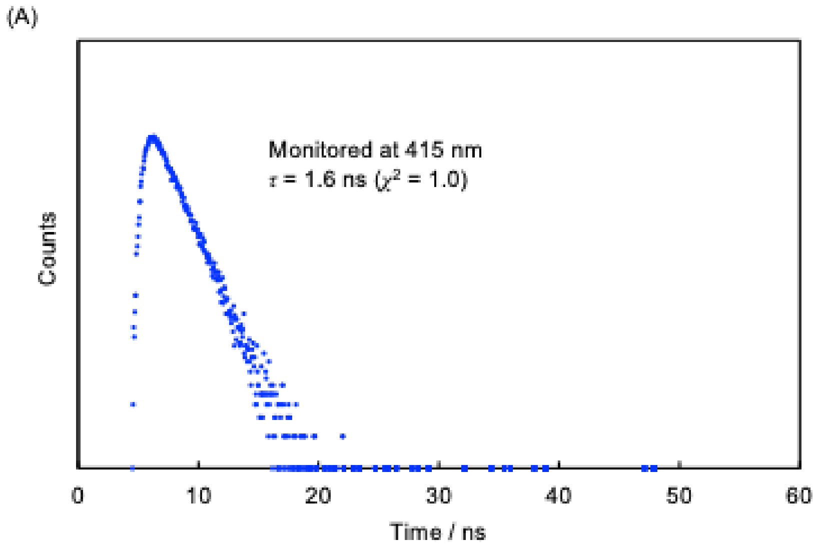

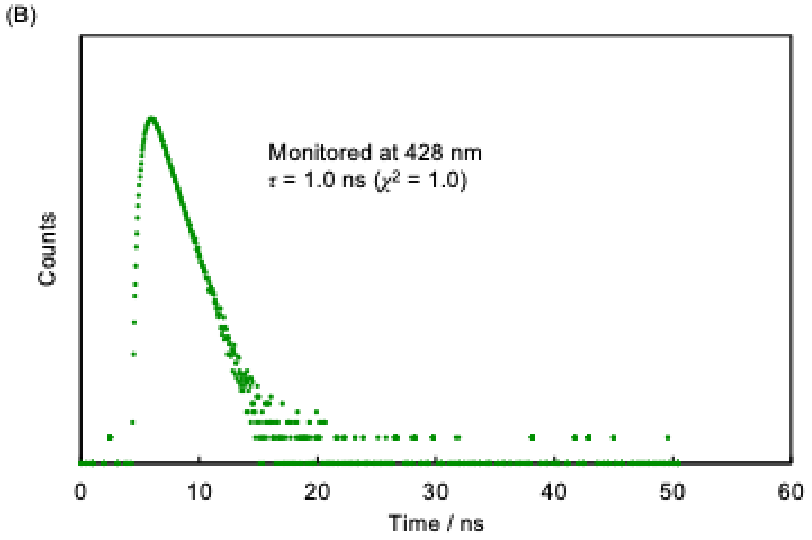

Figure S17: PL decay curves and the data of (A) (S_p)-**6** and (B) (S_p)-**7** in $CHCl_3$. The decay curves were fitted with a single exponential function.

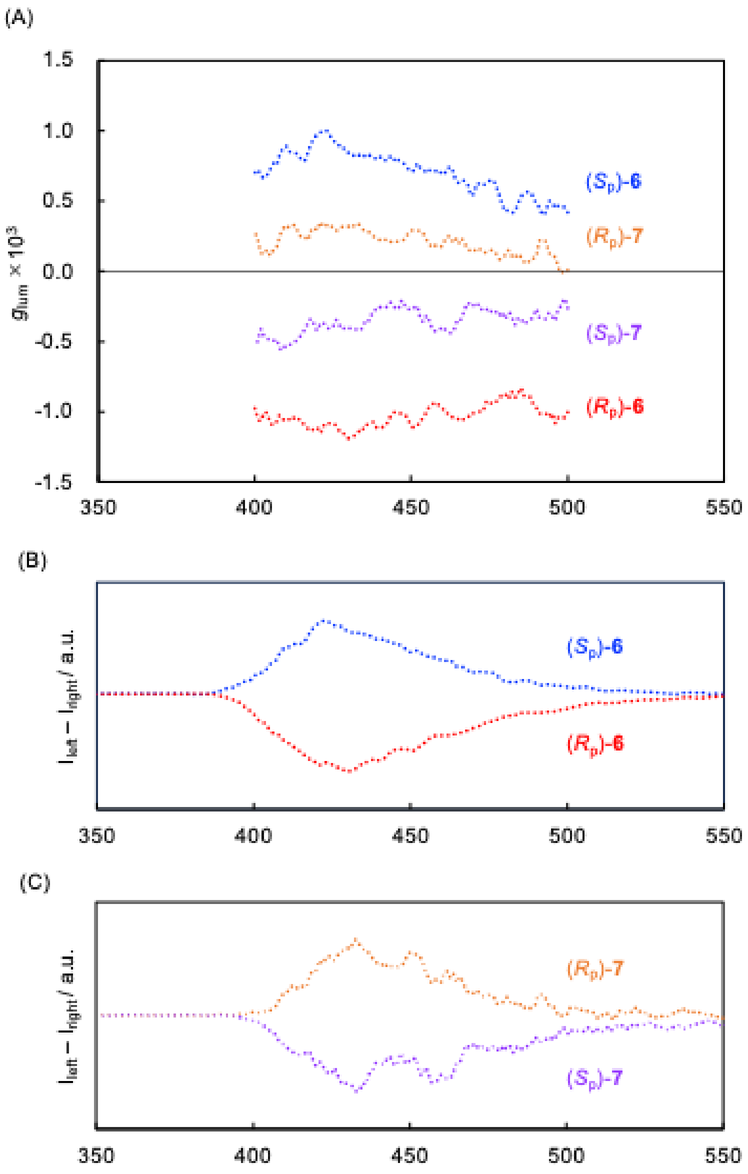

Figure S18: (A) The g_{lum} charts of **6** and **7** with the CPL spectra of (B) **6** and (C) **7** in $CHCl_3$ (1.0×10^{-5} M) excited at 300 nm.

(A)

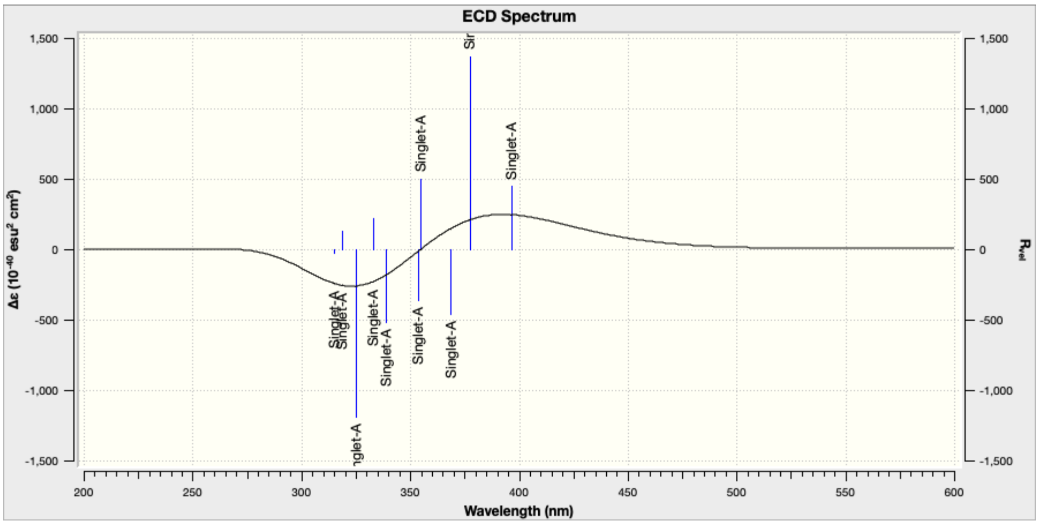

(B)

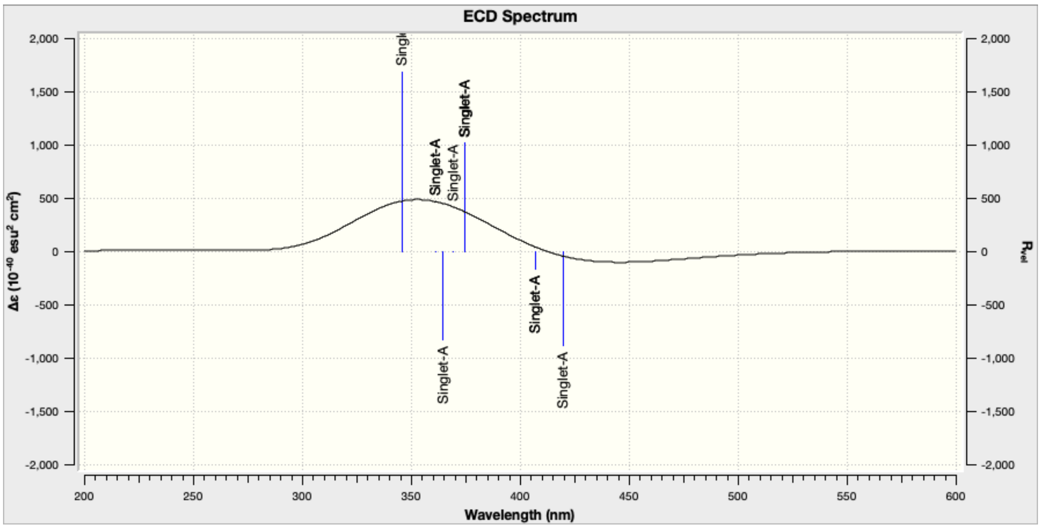

Figure S19: Calculated ECD spectrum of (A) (S_p)-**6** and (B) (S_p)-**7** simulated using TD-DFT calculation (TD-MN15/6-31G(d)//MN15/6-31G(d)).

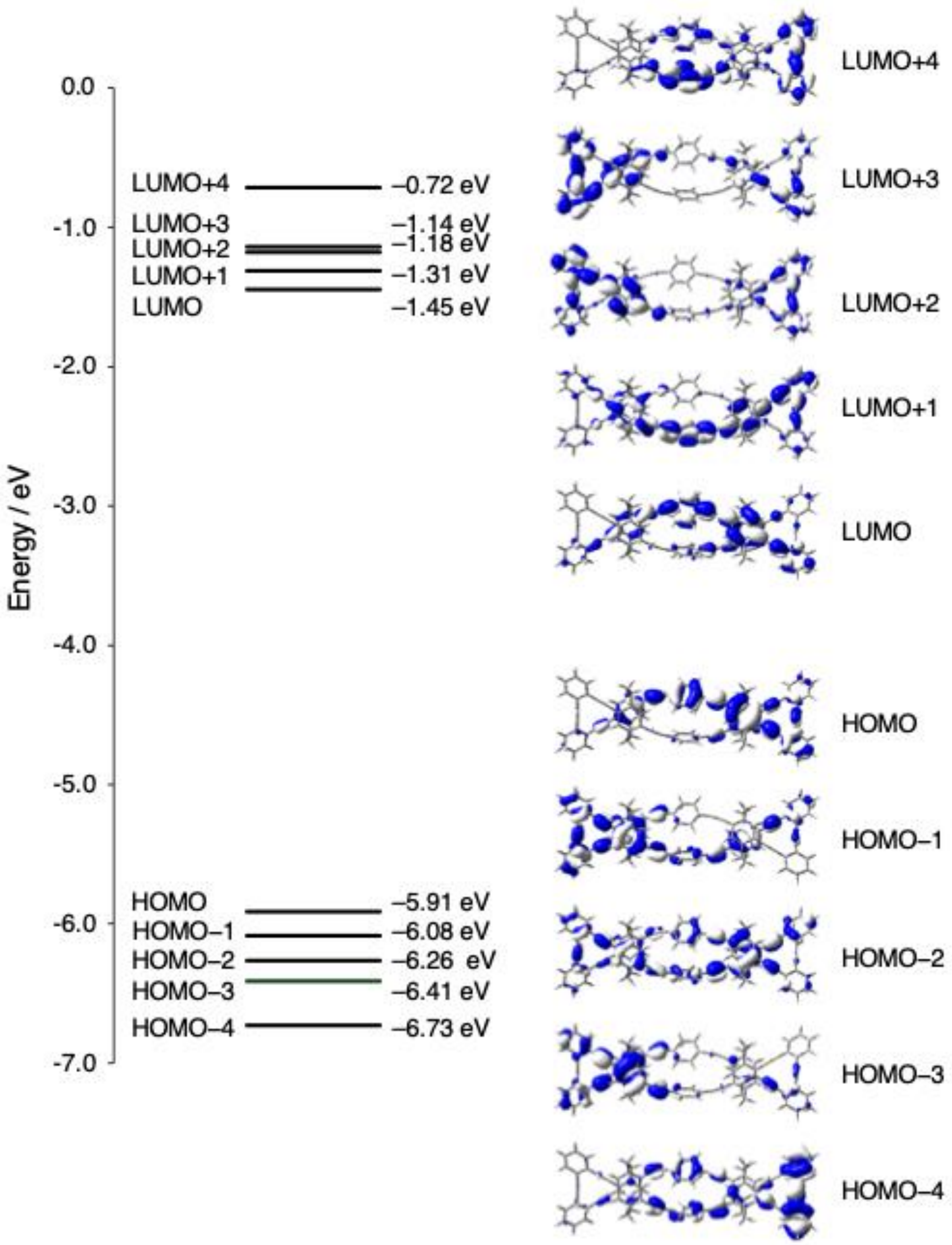

Figure S20: Molecular orbitals of (S_p)-**6** in the ground state simulated using DFT calculation (MN15/6-31G(d).

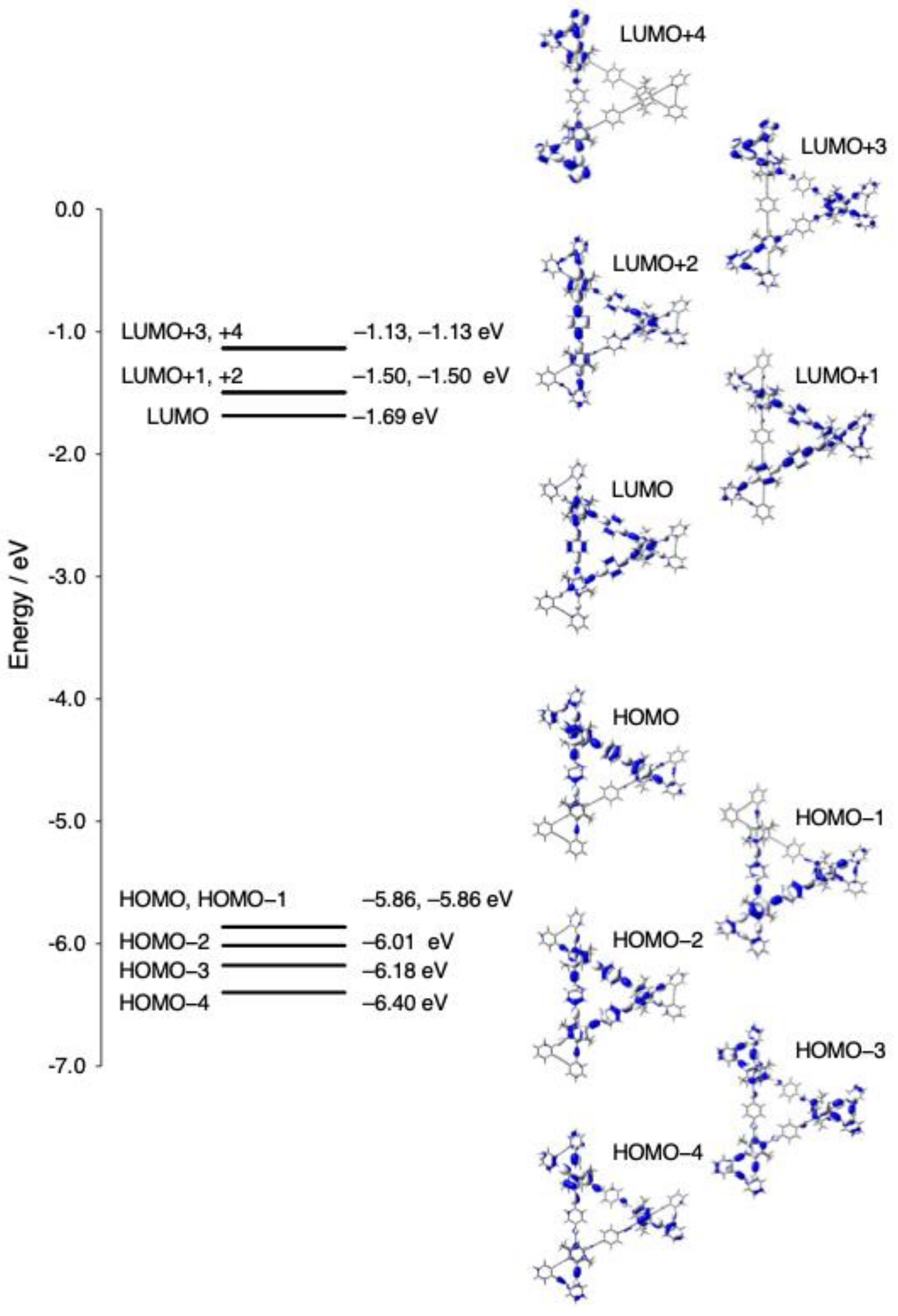

Figure S21: Molecular orbitals of (S_p)-**7** in the ground state simulated using DFT calculation (MN15/6-31G(d).

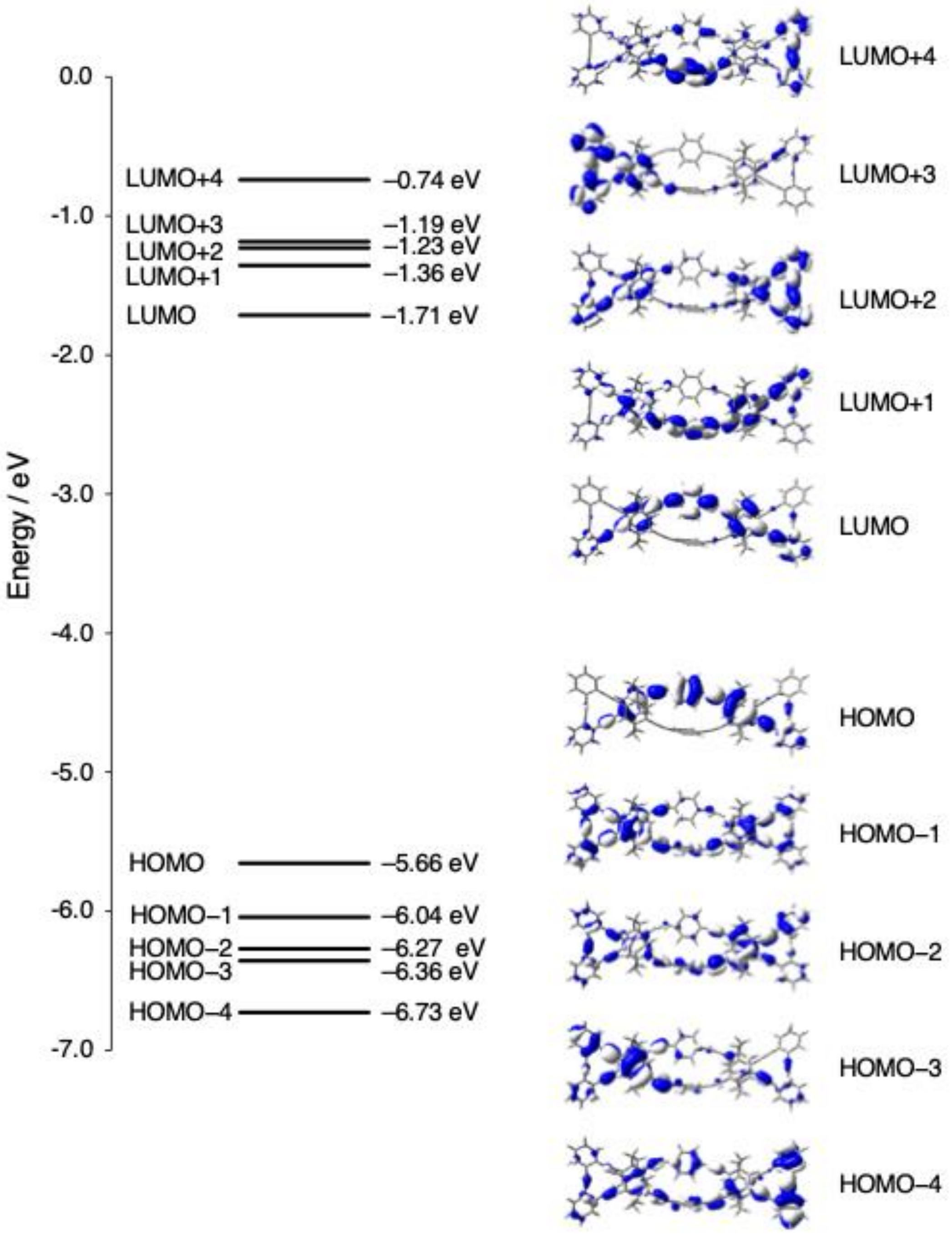

Figure S22: Molecular orbitals of (S_p)-**6** in the excited state simulated using DFT calculation TD-DFT calculation (TD-MN15/6-31G(d)//MN15/6-31G(d)).

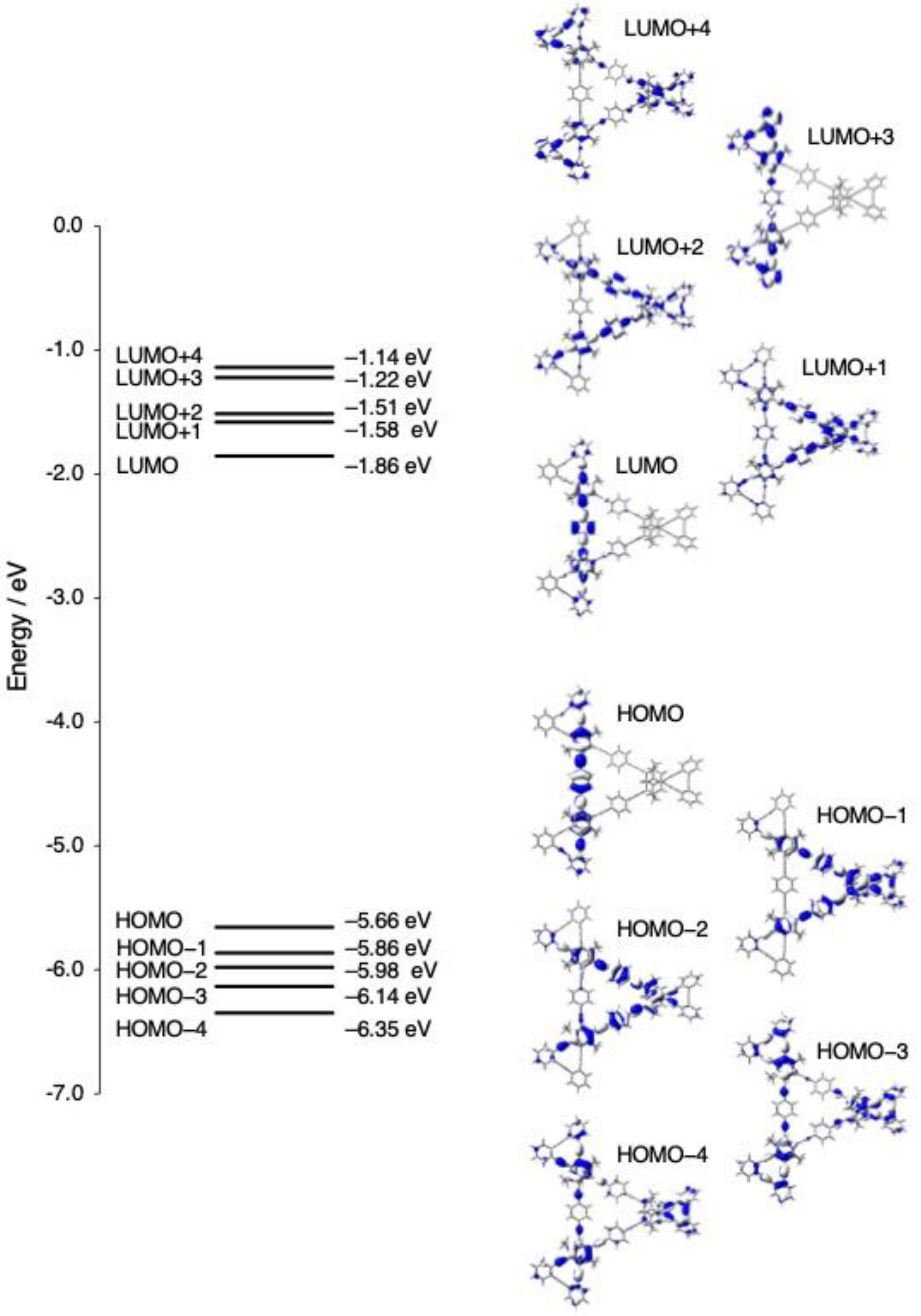

Figure S23: Molecular orbitals of (S_p)-**7** in the excited state simulated using DFT calculation TD-DFT calculation (TD-MN15/6-31G(d)//MN15/6-31G(d)).

Table S1: Cartesian coordinates of (S_p)-**6** in the ground state (TD-MN15/6-31G(d)).

atom	x	y	z
C	5.219175	-1.227128	-1.324986
C	-2.831549	2.227834	-0.690421
C	3.955595	-1.834561	-1.035434
C	-1.446275	2.312284	-0.335084
C	-0.399553	-2.770788	1.041687
H	-0.746589	-3.06038	2.029519
C	-1.335604	-2.338332	0.085558
C	-3.832914	-1.754375	0.736629
C	7.610024	-1.10228	-1.282332
H	8.581278	-1.531192	-1.044206
C	-3.977258	1.910872	-0.956755
C	5.179775	0.106672	-1.773978
H	4.204112	0.572072	-1.89942
C	1.416502	-2.348429	-0.51217
C	-2.70828	-2.114726	0.436964
C	1.306091	2.196819	0.241963
C	2.808573	-2.155349	-0.779989
C	7.567378	0.244098	-1.686104
C	0.95873	-2.780277	0.745301
H	1.682251	-3.078979	1.499572
C	-5.085446	-1.159591	1.103928
C	6.45079	-1.799326	-0.946118
C	-5.235683	1.309506	-1.281907
C	5.102883	1.140036	1.133637
C	6.344799	1.73973	0.837107
C	6.525224	-2.930391	0.053325
H	7.551572	-3.313194	0.069532
H	5.862006	-3.758197	-0.223542
C	-5.046831	0.164107	1.580748
H	-4.075871	0.64907	1.667538
C	-6.472748	1.862598	-0.895107
C	6.321839	0.899213	-1.806578
C	7.494247	1.067676	1.248041
H	8.469272	1.518767	1.075426
C	6.220287	-0.962975	1.686429
C	3.845536	1.723797	0.785464
C	5.06591	-0.1963	1.577207
H	4.093763	-0.682186	1.641327
C	2.701513	2.03974	0.509021
C	-6.326552	-1.747627	0.783169
C	-6.322725	-0.811356	-1.827977
C	7.45614	-0.279682	1.648983
C	-7.572894	-0.167157	-1.696135
C	-6.549983	2.953799	0.14783
H	-7.58196	3.318294	0.19594
H	-5.904423	3.802891	-0.105231
C	-5.186364	-0.013019	-1.760914
H	-4.206066	-0.469047	-1.890315
C	0.473502	-1.976647	-1.488902
H	0.825042	-1.648363	-2.462649
C	-7.626295	1.167371	-1.255285
H	-8.601651	1.581463	-1.008029
C	6.461448	2.872736	-0.155704
H	7.476004	3.282224	-0.098769
H	5.758767	3.682834	0.071613
C	6.190065	2.39651	-1.645871
H	5.169509	2.683765	-1.922763
H	6.881022	2.937166	-2.303125
C	-7.43772	0.247091	1.649754
C	-0.881258	-1.973866	-1.194931
H	-1.602765	-1.63646	-1.935029
C	-0.604156	3.382304	-0.678063
H	-1.018642	4.245919	-1.190413
C	-0.900878	1.200214	0.33461
H	-1.560492	0.380259	0.606106
C	-6.180228	-2.312555	-1.719534
H	-5.157673	-2.582395	-2.007323
H	-6.867238	-2.834603	-2.395624
C	-7.477008	-1.087386	1.210277
H	-8.451957	-1.53242	1.022376
C	-6.202817	0.929363	1.706187
C	6.135412	-2.462869	1.519193
H	5.103936	-2.774016	1.721453
H	6.788937	-2.988046	2.225474
C	-6.445225	-2.845627	-0.247886
H	-7.459317	-3.257712	-0.202539
H	-5.740777	-3.662663	-0.053746
C	0.452812	1.13821	0.612755
H	0.873261	0.262911	1.101877
C	0.755953	3.325468	-0.39038
H	1.407562	4.148426	-0.670434
C	-6.12537	2.434871	1.58664
H	-5.091507	2.743167	1.777493
H	-6.765685	2.932186	2.324725
C	-8.669894	0.956405	1.785936
C	-9.733902	1.546073	1.829285
C	8.6876	-0.993605	1.760307
C	9.750805	-1.586301	1.782426
C	8.786872	0.986466	-1.716311

C	9.8353	1.603217	-1.668262
C	-8.787737	-0.916135	-1.749562
C	-9.832882	-1.539417	-1.717192
C	-10.985037	2.23046	1.880164
C	-12.123538	1.702487	1.219156
C	-11.102067	3.437112	2.589608
C	-13.342922	2.394873	1.302128
C	-12.316172	4.110457	2.655326
H	-10.222431	3.830797	3.091283
C	-13.440569	3.586412	2.011444
H	-14.209285	1.981194	0.793714
H	-12.388141	5.042065	3.210075
H	-14.392328	4.108394	2.061511
C	-11.066145	-2.255946	-1.675676
C	-12.16446	-1.755054	-0.930759
C	-11.20683	-3.465893	-2.374983
C	-13.36957	-2.476547	-0.922329
C	-12.405836	-4.168488	-2.349565
H	-10.358196	-3.838909	-2.941626
C	-13.491232	-3.671076	-1.622925
H	-14.20513	-2.083238	-0.350169
H	-12.496665	-5.102285	-2.897852
H	-14.431095	-4.216065	-1.601776
C	-12.056396	0.486502	0.469782
C	-12.069853	-0.537034	-0.187635
C	11.070178	2.315964	-1.612252
C	12.171888	1.793046	-0.887852
C	11.208532	3.544926	-2.278118
C	13.377882	2.51282	-0.865811
C	12.408523	4.245221	-2.23976
H	10.357203	3.93451	-2.829375
C	13.497221	3.726384	-1.533312
H	14.216083	2.102683	-0.30956
H	12.497526	5.193892	-2.762196
H	14.437905	4.269473	-1.502097
C	11.001807	-2.271972	1.81004
C	12.138823	-1.724574	1.162274
C	11.121088	-3.498962	2.483415
C	13.358652	-2.418492	1.22208
C	12.335536	-4.173503	2.526402
H	10.242854	-3.90755	2.975551
C	13.458394	-3.630393	1.895719
H	14.223684	-1.989607	0.724088
H	12.409057	-5.120985	3.053396
H	14.410419	-4.153264	1.928276
C	12.080143	0.554569	-0.179096
C	12.069352	-0.487413	0.448704

Table S2: Cartesian coordinates of (S_p)-**6** in the S_1 state (TD-MN15/6-31G(d)).

atom	x	y	z
C	5.21876	-1.281031	-1.469605
C	-2.856091	2.390252	-0.848631
C	3.963628	-1.923135	-1.223429
C	-1.489565	2.438988	-0.526214
C	-0.289669	-2.704487	1.028702
H	-0.587103	-2.84692	2.063642
C	-1.271624	-2.400419	0.06845
C	-3.723341	-1.682827	0.75901
C	7.604974	-1.113999	-1.330765
H	8.573343	-1.531255	-1.061922
C	-4.006046	2.013959	-1.079566
C	5.1708	0.065297	-1.886048
H	4.19227	0.518714	-2.033314
C	1.450187	-2.500485	-0.653219
C	-2.621562	-2.105313	0.453556
C	1.267974	2.241748	0.089142
C	2.828107	-2.290245	-0.976688
C	7.554067	0.238716	-1.719033
C	1.05223	-2.760948	0.671263
H	1.813202	-2.946997	1.425567
C	-4.966549	-1.063092	1.116603
C	6.446586	-1.839759	-1.060519
C	-5.225966	1.362987	-1.342819
C	4.976111	1.077138	1.006352
C	6.252081	1.685943	0.770367
C	6.498706	-2.987788	-0.079726
H	7.532345	-3.345183	-0.013905
H	5.871769	-3.826675	-0.403741
C	-4.933965	0.284715	1.525456
H	-3.966973	0.784132	1.56604
C	-6.488227	1.897854	-0.966408
C	6.301994	0.874576	-1.873264
C	7.375367	1.006928	1.193191
H	8.359188	1.447946	1.046096
C	6.072922	-1.051009	1.552037
C	3.764749	1.679932	0.667002
C	4.931022	-0.29675	1.400174
H	3.957756	-0.785138	1.396253
C	2.61951	2.05452	0.3856
C	-6.207795	-1.677359	0.847467
C	-6.255971	-0.819782	-1.777419
C	7.325946	-0.361772	1.579052
C	-7.531612	-0.203201	-1.659903
C	-6.563635	3.013568	0.04792
H	-7.605571	3.340585	0.133576
H	-5.961016	3.879781	-0.250903
C	-5.144839	0.002783	-1.743561
H	-4.151461	-0.432059	-1.849602
C	0.460241	-2.27901	-1.629036
H	0.762352	-2.089144	-2.654822
C	-7.618083	1.153742	-1.268066
H	-8.601454	1.539879	-1.007096
C	6.389993	2.83132	-0.201274
H	7.405229	3.234978	-0.120917
H	5.685084	3.641727	0.020577
C	6.143319	2.36798	-1.699344
H	5.12229	2.642721	-1.987506
H	6.834854	2.926781	-2.340744
C	-7.325794	0.339849	1.648755
C	-0.879567	-2.234791	-1.27338
H	-1.637712	-2.00941	-2.01988
C	-0.674694	3.602093	-0.485272
H	-1.119503	4.569506	-0.700934
C	-0.892077	1.178268	-0.234564
H	-1.531283	0.300124	-0.247502
C	-6.080562	-2.313952	-1.627923
H	-5.051863	-2.567846	-1.909236
H	-6.755829	-2.869134	-2.289599
C	-7.35813	-1.012016	1.26355
H	-8.330816	-1.475563	1.112088
C	-6.099074	1.039241	1.647284
C	6.017518	-2.548541	1.366182
H	4.980292	-2.875336	1.505622
H	6.637786	-3.072688	2.103082
C	-6.332483	-2.810484	-0.142972
H	-7.345113	-3.223447	-0.076798
H	-5.62298	-3.618939	0.067836
C	0.435867	1.08029	0.084603
H	0.878953	0.117073	0.325125
C	0.671845	3.503055	-0.206088
H	1.295932	4.392323	-0.197292
C	-6.054308	2.541849	1.476107
H	-5.017791	2.87301	1.604666
H	-6.665053	3.050868	2.231372
C	-8.565601	1.035498	1.785962
C	-9.639919	1.60684	1.82586
C	8.535203	-1.060337	1.733486
C	9.614224	-1.642939	1.785642
C	8.761738	0.999174	-1.694882
C	9.796818	1.631977	-1.588155
C	-8.716902	-0.979697	-1.669645

C	-9.751725	-1.624654	-1.594367
C	-10.899826	2.275551	1.869109
C	-12.038775	1.713573	1.236313
C	-11.024475	3.502228	2.54186
C	-13.265603	2.394472	1.311022
C	-12.245828	4.163287	2.599439
H	-10.144599	3.921515	3.021932
C	-13.370315	3.606239	1.983879
H	-14.132059	1.955059	0.824846
H	-12.323279	5.11082	3.125745
H	-14.327746	4.118341	2.027654
C	-10.967734	-2.352459	-1.501487
C	-12.072522	-1.820653	-0.781745
C	-11.097729	-3.607455	-2.125795
C	-13.270868	-2.551988	-0.732295
C	-12.289139	-4.316402	-2.057943
H	-10.246056	-4.005339	-2.670574
C	-13.381667	-3.78569	-1.362496
H	-14.109398	-2.135207	-0.181376
H	-12.371832	-5.282041	-2.549529
H	-14.316381	-4.337295	-1.309831
C	-11.967188	0.475378	0.526051
C	-11.982632	-0.571387	-0.09513
C	11.010318	2.37067	-1.459843
C	12.081565	1.871202	-0.672543
C	11.15789	3.603746	-2.116014
C	13.268429	2.620061	-0.584179
C	12.337091	4.331766	-2.010107
H	10.330357	3.974048	-2.714802
C	13.396487	3.836339	-1.243579
H	14.083373	2.228754	0.018255
H	12.433462	5.283041	-2.526412
H	14.321149	4.400981	-1.159564
C	10.865594	-2.292445	1.850842
C	12.036638	-1.669075	1.325656
C	10.980554	-3.566884	2.449276
C	13.273628	-2.324884	1.452445
C	12.209939	-4.19619	2.552239
H	10.081874	-4.036475	2.83975
C	13.364636	-3.570949	2.058138
H	14.159169	-1.838909	1.052223
H	12.278674	-5.173734	3.022073
H	14.330007	-4.062608	2.141593
C	11.982404	0.631241	0.026872
C	11.972375	-0.418112	0.646725

Table S3: Cartesian coordinates of (S_p)-**7** in the ground state (MN15/6-31G(d)).

atom	x	y	z
C	5.645152	7.194323	1.658074
C	5.444349	5.796838	1.687073
C	4.130192	5.337312	1.646262
C	3.32606	7.541392	0.98561
C	4.57706	8.046849	1.325473
H	3.937351	4.267908	1.707323
H	4.801005	9.09625	1.144126
C	3.803161	6.984713	-1.687613
C	5.06827	7.611627	-1.659545
C	6.211571	6.862991	-1.326962
C	6.121903	5.517043	-0.985745
C	3.776543	5.592826	-1.645509
H	7.138847	7.403297	-1.146822
H	2.820563	5.07611	-1.705543
C	7.136992	4.916406	-0.040754
H	8.034662	5.544219	-0.049916
H	7.429326	3.904516	-0.345198
C	6.586654	4.835993	1.445098
H	7.42631	5.026759	2.123881
H	6.22866	3.817341	1.631329
C	2.533796	7.770225	-1.445289
H	1.677653	7.112325	-1.631335
H	2.452948	8.627282	-2.124327
C	2.438843	8.318367	0.040465
H	2.75606	9.366879	0.049279
H	1.38633	8.281125	0.345151
C	5.183712	9.034293	-1.699879
C	5.320873	10.243069	-1.655059
C	6.961026	7.747268	1.697167
C	8.06747	8.25283	1.651439
C	5.496338	11.657948	-1.600927
C	4.559172	12.511303	-2.20633
C	6.767738	13.616035	-0.915338
C	4.722624	13.891015	-2.16842
H	3.704866	12.068406	-2.710689
H	7.630755	14.036089	-0.406447
H	3.988167	14.535534	-2.643828
C	9.358811	8.8568	1.595501
C	10.460158	8.230332	2.201662
C	9.545862	10.095787	0.930599
C	11.721676	8.812341	2.162634
H	10.303143	7.281743	2.707533
C	10.827478	10.670394	0.906859
H	12.561849	8.313967	2.638612
H	10.959975	11.620383	0.396765
C	4.913673	4.847555	-1.285104
C	3.067027	6.184868	1.286283
C	5.831589	14.44494	-1.522742
C	11.905334	10.037323	1.515123
C	6.617683	12.219534	-0.93802
C	7.595027	11.399788	-0.291167
C	8.463356	10.770602	0.283749
H	12.889392	10.497168	1.483627
H	5.964542	15.522985	-1.491962
C	4.776176	3.456133	-0.992933
C	1.787519	5.620001	0.996041
C	0.706634	5.140871	0.703373
C	4.659679	2.28015	-0.697804
C	3.407507	-8.491934	1.659839
C	2.295405	-7.622082	1.689609
C	2.551329	-6.253347	1.654246
C	4.864706	-6.651001	0.997436
C	4.67945	-7.988816	1.332104
H	1.719475	-5.554157	1.715432
H	5.478295	-8.704851	1.149156
C	4.148317	-6.775526	-1.677817
C	4.062417	-8.184839	-1.656104
C	2.843345	-8.805295	-1.329022
C	1.719718	-8.059038	-0.987565
C	2.954113	-6.059922	-1.636428
H	2.850196	-9.879079	-1.152345
H	2.98174	-4.973336	-1.692155
C	0.691961	-8.644061	-0.046598
H	0.789087	-9.735086	-0.060262
H	-0.33052	-8.392382	-0.351855
C	0.893794	-8.133072	1.441972
H	0.639726	-8.959009	2.116813
H	0.188359	-7.316103	1.630392
C	5.461277	-6.067057	-1.430125
H	5.317611	-4.99617	-1.611783
H	6.245906	-6.420973	-2.109311
C	5.981733	-6.264267	0.055636
H	6.733385	-7.061138	0.062078
H	6.472811	-5.334152	0.365155
C	5.239344	-8.992384	-1.69703
C	6.220709	-9.711346	-1.652886
C	3.232047	-9.908617	1.693479
C	3.120296	-11.119793	1.642888
C	7.361571	-10.566353	-1.598655
C	8.569083	-10.174042	-2.19943
C	8.428318	-12.644891	-0.918807
C	9.685537	-11.00101	-2.161974
H	8.609912	-9.21059	-2.699902
H	8.363206	-13.604661	-0.41401

H	10.610826	-10.681518	-2.633778	H	-10.282427	-0.387749	1.155569
C	3.003507	-12.540319	1.579088	C	-7.953635	-0.209492	-1.676499
C	1.910801	-13.18837	2.178205	C	-9.129442	0.572465	-1.654365
C	3.988021	-13.314579	0.913115	C	-9.053439	1.939122	-1.330106
C	1.789426	-14.572154	2.131162	C	-7.843401	2.536428	-0.991276
H	1.163801	-12.583695	2.684908	C	-6.735224	0.464162	-1.638889
C	3.850258	-14.712211	0.881471	H	-9.984919	2.473307	-1.153124
H	0.937935	-15.056325	2.601661	H	-5.809421	-0.105278	-1.695317
H	4.610474	-15.296265	0.370431	C	-7.832599	3.721023	-0.052741
C	1.740849	-6.676741	-1.281625	H	-8.825581	4.183464	-0.06537
C	3.816524	-5.752156	1.298493	H	-7.102905	4.479144	-0.360695
C	9.614144	-12.241313	-1.521637	C	-7.489051	3.293313	1.436012
C	2.763452	-15.336503	1.482704	H	-8.074811	3.928767	2.110746
C	7.29069	-11.821078	-0.940821	H	-6.428121	3.494256	1.621814
C	6.09214	-12.264437	-0.298613	C	-7.998865	-1.700271	-1.425619
C	5.112857	-12.707007	0.271929	H	-7.000826	-2.113378	-1.609088
H	2.67379	-16.418789	1.444887	H	-8.700246	-2.202845	-2.102128
H	10.483858	-12.892074	-1.491326	C	-8.426267	-2.048799	0.061944
C	0.60207	-5.865665	-0.988702	H	-9.492565	-2.299611	0.071979
C	3.965726	-4.360917	1.011441	H	-7.866846	-2.939382	0.371673
C	4.093756	-3.185518	0.71888	C	-10.418689	-0.04015	-1.692424
C	-0.359513	-5.179301	-0.692655	C	-11.533252	-0.527538	-1.645312
C	4.235902	-1.808525	0.368214	C	-10.19756	2.160159	1.691994
C	5.512396	-1.245474	0.183773	C	-11.189499	2.863839	1.639454
C	3.101981	-0.993651	0.19055	C	-12.84507	-1.08561	-1.587943
C	5.650878	0.089237	-0.166318	C	-13.111846	-2.329067	-2.184188
H	6.389796	-1.871453	0.321233	C	-15.177755	-0.963617	-0.906369
C	3.240428	0.339872	-0.162886	C	-14.387259	-2.879953	-2.143793
H	2.115179	-1.425952	0.334886	H	-12.299265	-2.849436	-2.683509
C	4.517244	0.903398	-0.346153	H	-15.974931	-0.424253	-0.402572
H	6.637986	0.520881	-0.307195	H	-14.575359	-3.842299	-2.612129
H	2.363583	0.966687	-0.302976	C	-12.358746	3.679055	1.577713
C	-1.481001	-4.367297	-0.342769	C	-12.367781	4.949849	2.176034
C	-1.332259	-2.97821	-0.170053	C	-13.524603	3.217113	0.914813
C	-2.751365	-4.942678	-0.154082	C	-13.502827	5.750704	2.13129
C	-2.418046	-2.190844	0.181476	H	-11.468222	5.291578	2.680417
H	-0.35223	-2.531594	-0.316841	C	-14.663391	4.039072	0.885452
C	-3.838083	-4.154646	0.194183	H	-13.491937	6.730428	2.601229
H	-2.8699	-6.014536	-0.286822	H	-15.551826	3.675358	0.376903
C	-3.689154	-2.766071	0.367983	C	-6.659046	1.823778	-1.286249
H	-2.300408	-1.118894	0.317499	C	-6.894204	-0.429378	1.297464
H	-4.817614	-4.602142	0.338428	C	-15.423919	-2.193874	-1.504927
C	-9.059435	1.298455	1.659781	C	-14.654946	5.29281	1.485944
C	-7.749221	1.824792	1.686713	C	-13.894456	-0.392737	-0.931426
C	-6.693195	0.917287	1.650183	C	-13.676105	0.868435	-0.293229
C	-8.198114	-0.886494	1.000545	C	-13.566527	1.938651	0.275068
C	-9.262107	-0.054925	1.335501	H	-15.545435	5.914536	1.44996
H	-5.67115	1.28686	1.70848	H	-16.423105	-2.61976	-1.472411

C	-5.385985	2.403476	-0.997489
C	-5.764584	-1.255238	1.01018
C	-4.810191	-1.953449	0.718022
C	-4.310489	2.894766	-0.705079
C	-0.557315	4.57482	0.354864
C	-1.687374	5.396472	0.186303
C	-0.692177	3.186885	0.16355
C	-2.912807	4.848123	-0.161384
H	-1.58697	6.46821	0.334004
C	-1.916525	2.639006	-0.187312
H	0.178978	2.55034	0.295268
C	-3.047135	3.460663	-0.354562
H	-3.783392	5.485333	-0.290278
H	-2.017729	1.567324	-0.338019

Table S4: Cartesian coordinates of (S_p)-**7** in the S_1 state (TD-MN15/6-31G(d)).

atom	x	y	z
C	-9.118809	0.18091	1.688508
C	-7.867816	0.829004	1.784205
C	-6.72618	0.041434	1.655009
C	-8.045273	-1.819215	0.795297
C	-9.18622	-1.140894	1.211916
H	-5.74614	0.502422	1.762873
H	-10.168793	-1.550888	0.986714
C	-7.868785	-0.820631	-1.784177
C	-9.119079	-0.171211	-1.688412
C	-9.18506	1.150667	-1.211809
C	-8.043367	1.82778	-0.795268
C	-6.726304	-0.034274	-1.655054
H	-10.167184	1.561698	-0.986541
H	-5.746762	-0.496304	-1.762984
C	-8.153259	2.892372	0.271795
H	-9.189684	3.245532	0.305078
H	-7.509304	3.753494	0.05791
C	-7.758577	2.335289	1.703769
H	-8.401623	2.827194	2.443177
H	-6.722461	2.620188	1.917236
C	-7.761144	-2.32703	-1.703748
H	-6.725339	-2.61303	-1.917256
H	-8.40474	-2.818252	-2.443132
C	-8.156365	-2.883689	-0.27176
H	-9.193173	-3.235727	-0.304995
H	-7.513333	-3.745507	-0.057906
C	-10.338198	-0.904561	-1.808719
C	-11.396748	-1.505614	-1.827545
C	-10.337131	0.915565	1.8089
C	-11.395033	1.517756	1.827807
C	-12.644503	-2.197107	-1.842848
C	-12.786651	-3.386233	-2.576954
C	-14.974213	-2.389848	-1.167391
C	-13.999257	-4.064711	-2.607064
H	-11.927909	-3.761945	-3.126296
H	-15.819602	-1.994182	-0.611521
H	-14.090962	-4.982349	-3.181842
C	-12.642046	2.210582	1.843185
C	-12.782881	3.399864	2.577292
C	-13.75381	1.707231	1.120026
C	-13.994763	4.079632	2.607482
H	-11.923703	3.774667	3.126575
C	-14.971595	2.405799	1.167882
H	-14.085454	4.997369	3.182263
H	-15.817442	2.011033	0.61207
C	-6.79118	1.278237	-1.153448
C	-6.792479	-1.271005	1.153396
C	-15.097101	-3.563641	-1.901917
C	-15.093186	3.579725	1.902411
C	-13.755681	-1.692576	-1.119614
C	-13.664262	-0.495469	-0.342377
C	-13.663708	0.510024	0.34279
H	-16.043228	4.106734	1.924521
H	-16.047704	-4.08964	-1.923965
C	-5.586259	1.953142	-0.788634
C	-5.588313	-1.947197	0.788487
C	-4.572444	-2.519906	0.435596
C	-4.569734	2.524754	-0.435854
C	5.066071	7.541095	1.612354
C	4.951137	6.13323	1.601592
C	3.672495	5.591596	1.698722
C	2.660477	7.767302	1.260867
C	3.914691	8.336576	1.458885
H	3.552364	4.510269	1.727107
H	4.050432	9.405111	1.303847
C	2.794178	7.36024	-1.46636
C	4.023714	8.074053	-1.565141
C	5.250196	7.378562	-1.415602
C	5.284899	6.025081	-1.14191
C	2.847576	5.979781	-1.48581
H	6.156048	7.973329	-1.315857
H	1.924429	5.406243	-1.434765
C	6.445288	5.441866	-0.37271
H	7.298577	6.122535	-0.464722
H	6.747404	4.464456	-0.768612
C	6.103904	5.257346	1.166087
H	7.01452	5.471455	1.737935
H	5.833179	4.210169	1.343715
C	1.530384	8.061229	-1.025804
H	0.690233	7.366734	-1.137474
H	1.32219	8.944849	-1.640862
C	1.609046	8.529528	0.487613
H	1.862798	9.595027	0.513587
H	0.61257	8.408975	0.928724
C	4.050982	9.485737	-1.552957
C	4.128185	10.703966	-1.465626
C	6.341615	8.173327	1.506689
C	7.403724	8.74349	1.334072
C	4.239058	12.113042	-1.366862
C	3.168042	12.941941	-1.757376
C	5.523367	14.113543	-0.828509
C	3.275697	14.323021	-1.689959
H	2.258598	12.473497	-2.12333
H	6.441557	14.560503	-0.457673

H	2.440978	14.945816	-2.000285	H	6.146907	-7.979925	1.316143
C	8.645222	9.415095	1.127042	C	4.944589	-6.138381	-1.601442
C	9.845629	8.82962	1.561968	C	5.058043	-7.546366	-1.612291
C	8.684844	10.678676	0.481658	C	3.905791	-8.340646	-1.459138
C	11.062787	9.474047	1.374444	C	2.652137	-7.770066	-1.261331
H	9.801331	7.862259	2.054586	C	3.666539	-5.595391	-1.698788
C	9.924988	11.316551	0.307298	H	4.040369	-9.409338	-1.304155
H	11.980882	9.006055	1.719275	H	3.547555	-4.513936	-1.727106
H	9.944435	12.284352	-0.185846	C	1.599737	-8.531254	-0.48837
C	4.074854	5.279576	-1.313084	H	1.852385	-9.597014	-0.51438
C	2.522569	6.383896	1.514638	H	0.603486	-8.409629	-0.929698
C	4.459525	14.912605	-1.228602	C	1.521219	-8.063013	1.025069
C	11.102042	10.722665	0.747115	H	1.311967	-8.94647	1.640002
C	5.435	12.712145	-0.880122	H	0.681767	-7.367654	1.13661
C	6.532847	11.923309	-0.421988	C	6.098175	-5.263735	-1.165624
C	7.497739	11.31652	0.006893	H	5.828595	-4.216264	-1.343261
H	12.051009	11.231236	0.600012	H	7.008704	-5.478776	-1.73726
H	4.547222	15.99445	-1.177416	C	6.438983	-5.448693	0.373248
C	4.057902	3.902964	-1.06757	H	7.291547	-6.130247	0.46543
C	1.263162	5.740345	1.323892	H	6.742007	-4.471618	0.769282
C	0.20861	5.180714	1.07885	C	6.332906	-8.17994	-1.506425
C	4.0462	2.705614	-0.767651	C	7.394394	-8.751211	-1.333653
C	-1.003789	4.511633	0.736482	C	4.040231	-9.49017	1.552657
C	-2.183997	5.240573	0.496117	C	4.116161	-10.708473	1.465265
C	-1.030886	3.110067	0.598208	C	8.635129	-9.424164	-1.126423
C	-3.349696	4.591292	0.119324	C	9.836263	-8.839979	-1.561074
H	-2.167294	6.321742	0.604169	C	9.912663	-11.327032	-0.306467
C	-2.195664	2.462123	0.217807	C	11.052683	-9.485732	-1.373308
H	-0.122593	2.544617	0.790341	H	9.793124	-7.872555	-2.05367
C	-3.373409	3.191956	-0.032881	H	9.930952	-12.294869	0.18665
H	-4.256988	5.157756	-0.071296	H	11.971358	-9.018723	-1.71793
H	-2.213042	1.380964	0.106959	C	4.22557	-12.11766	1.366405
C	4.039922	1.359804	-0.396796	C	3.153616	-12.945471	1.756644
C	2.814332	0.655772	-0.198567	C	5.420979	-12.717966	0.879846
C	5.264283	0.657456	-0.186458	C	3.259845	-14.326659	1.689131
C	2.813468	-0.658632	0.198428	H	2.244593	-12.476111	2.122468
H	1.879428	1.185837	-0.362377	C	5.507896	-14.119449	0.828126
C	5.263415	-0.663369	0.186834	H	2.424418	-14.948609	1.999249
H	6.200065	1.189904	-0.335617	H	6.425691	-14.567334	0.45743
C	4.038135	-1.364191	0.396912	C	2.515741	-6.386491	-1.514999
H	1.877864	-1.187523	0.362042	C	4.068498	-5.284024	1.313103
H	6.198497	-1.196992	0.336191	C	11.090456	-10.734405	-0.746004
C	4.014418	-8.078456	1.564939	C	4.443144	-14.917437	1.227948
C	2.785642	-7.36337	1.465964	C	8.673248	-10.687811	-0.481082
C	2.840464	-5.982965	1.485539	C	7.485358	-11.324413	-0.006609
C	5.277809	-6.030761	1.142131	C	6.519744	-11.930231	0.422016
C	5.241651	-7.384225	1.415718	H	4.529722	-15.999368	1.176685
H	1.917923	-5.408465	1.434364	H	12.038841	-11.244003	-0.598707

C	1.256993	-5.741607	-1.32441
C	4.053037	-3.907378	1.067654
C	4.042712	-2.710013	0.767753
C	0.203029	-5.180839	-1.079434
C	-3.376856	-3.188342	0.032492
C	-3.354652	-4.587687	-0.11987
C	-2.198329	-2.459756	-0.218149
C	-2.189661	-5.238188	-0.496744
H	-4.262553	-5.15319	0.070705
C	-1.034258	-3.108918	-0.598638
H	-2.214535	-1.37859	-0.107184
C	-1.008667	-4.5105	-0.737045
H	-2.174127	-6.319363	-0.604902
H	-0.125357	-2.544432	-0.790731
